# Supplementary material for: The Chlamydia trachomatis inclusion membrane protein CT006 associates with lipid droplets in eukaryotic cells
Source: PLoS One. 2022 Feb 22;17(2):e0264292. doi: 10.1371/journal.pone.0264292 (PMC8863265; doi:10.1371/journal.pone.0264292)
Supplement: S1 Fig — Whole cell extracts from S. cerevisiae NSY01 producing the indicated Inc fragments fused to GFP were analyzed by immunoblotting using antibodies against GFP and PGK1 (yeast loading control) and appropriate HRP-conjugated secondary antibodies. (a) Proteins were detected using SuperSignal West Pico detection kit (Thermo Fisher Scientific). (b) Proteins were detected using SuperSignal West Femto detection kit (Thermo Fisher Scientific). * Represents proteins that migrated according to the predicted molecular mass; # represents proteins that migrated below the predicted molecular mass; the cross in (a) corresponds to a fusion protein that was not analyzed in this study. (PDF) [file pone.0264292.s001.pdf]

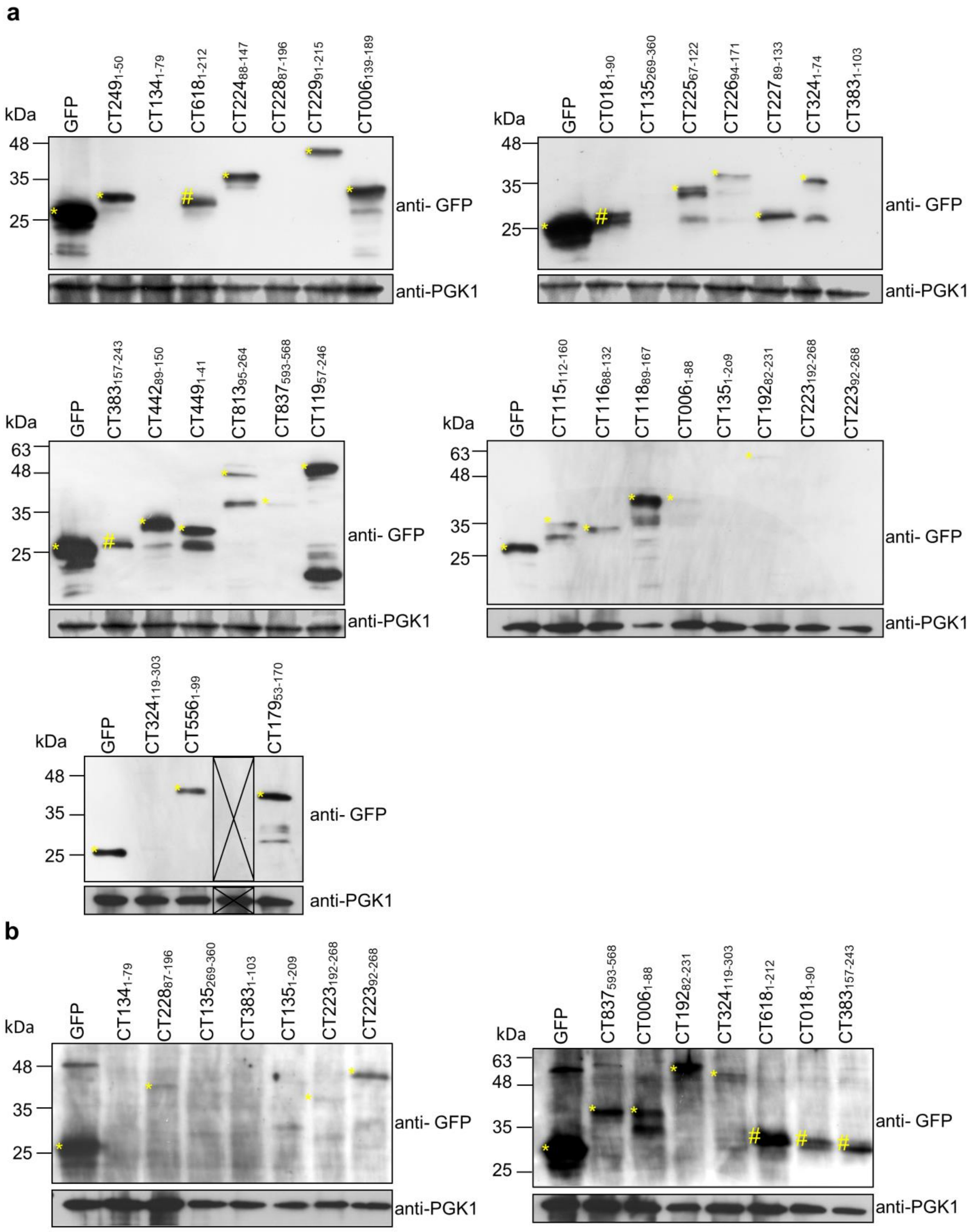

S1 Fig. Analysis of the production of CT006-GFP fusion proteins in yeast by immunoblotting. Whole cell extracts from *S. cerevisiae* NSY01 producing the indicated Inc fragments fused to GFP were analyzed by immunoblotting using antibodies against GFP and PGK1 (yeast loading control) and appropriate HRP-conjugated secondary antibodies. (a) Proteins were detected using SuperSignal West Pico detection kit (Thermo Fisher Scientific). (b) Proteins were detected using SuperSignal West Femto detection kit (Thermo Fisher Scientific). * Represents proteins that migrated according to the predicted molecular mass; # represents proteins that migrated below the predicted molecular mass; the cross in (a) corresponds to a fusion protein that was not analyzed in this study.
