# Supplementary material for: The Chlamydia trachomatis inclusion membrane protein CT006 associates with lipid droplets in eukaryotic cells
Source: PLoS One. 2022 Feb 22;17(2):e0264292. doi: 10.1371/journal.pone.0264292 (PMC8863265; doi:10.1371/journal.pone.0264292)
Supplement: S2 Fig — Whole cell extracts from S. cerevisiae NSY01 producing the indicated Inc fragments fused to GFP-Pep12L-TM were analyzed by immunoblotting using antibodies against GFP and PGK1 (yeast loading control) and appropriate HRP-conjugated secondary antibodies. (a) Proteins were detected using SuperSignal West Pico detection kit (Thermo Fisher Scientific). (b) Proteins were detected using SuperSignal West Femto detection kit (Thermo Fisher Scientific). * Represents proteins that migrated according to the predicted molecular mass; # represents proteins that migrated below the predicted molecular mass. (PDF) [file pone.0264292.s002.pdf]

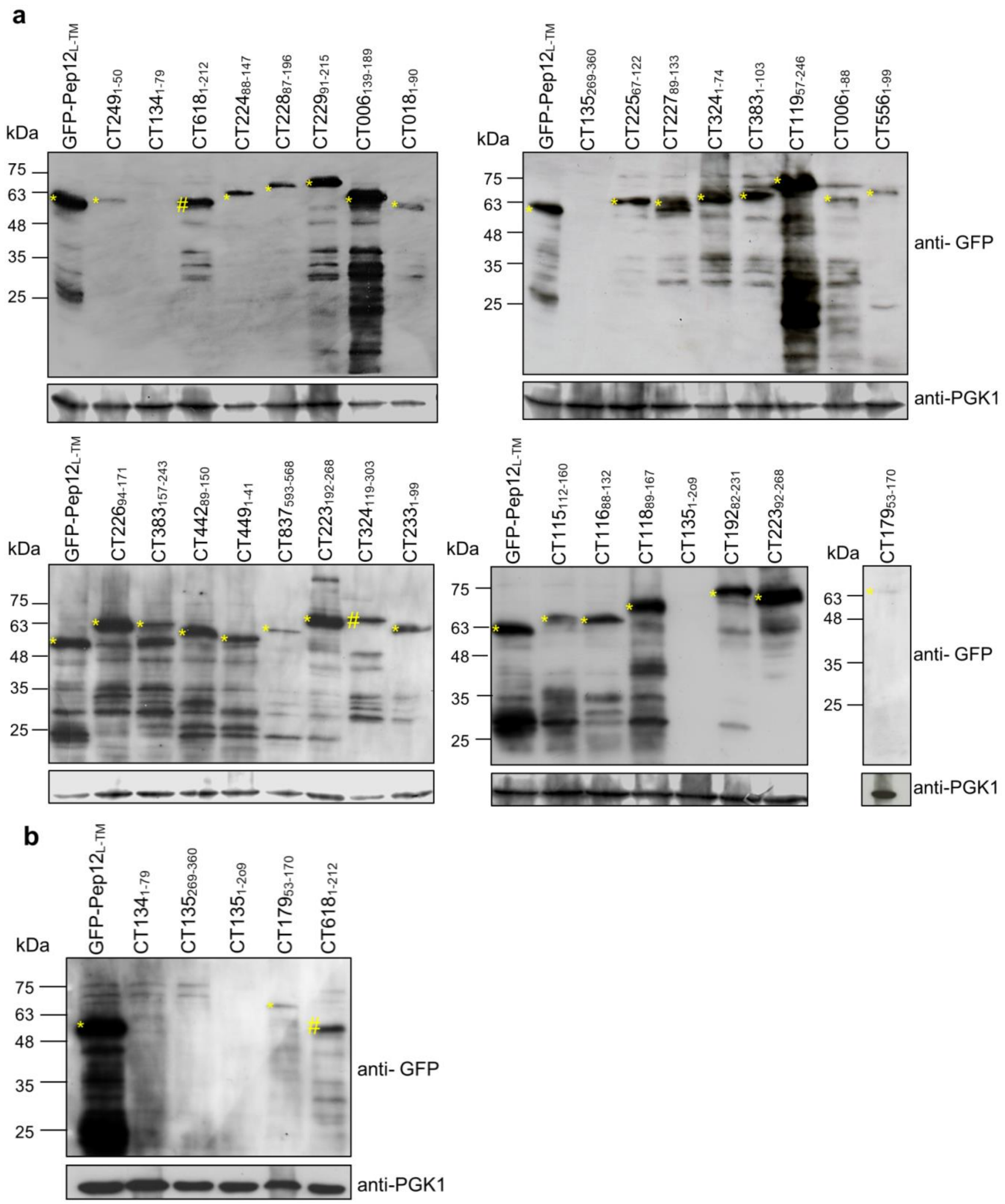

S2 Fig. Analysis of the production of Inc-GFP-Pep12$_{L-TM}$ fusion proteins in yeast by immunoblotting. Whole cell extracts from *S. cerevisiae* NSY01 producing the indicated Inc fragments fused to GFP-Pep12$_{L-TM}$ were analyzed by immunoblotting using antibodies against GFP and PGK1 (yeast loading control) and appropriate HRP-conjugated secondary antibodies. (a) Proteins were detected using SuperSignal West Pico detection kit (Thermo Fisher Scientific). (b) Proteins were detected using SuperSignal West Femto detection kit (Thermo Fisher Scientific). * Represents proteins that migrated according to the predicted molecular mass; # represents proteins that migrated below the predicted molecular mass.
