# Supplementary material for: The Chlamydia trachomatis inclusion membrane protein CT006 associates with lipid droplets in eukaryotic cells
Source: PLoS One. 2022 Feb 22;17(2):e0264292. doi: 10.1371/journal.pone.0264292 (PMC8863265; doi:10.1371/journal.pone.0264292)
Supplement: S3 Fig — S. cerevisiae NSY01 strains producing the indicated Inc fragments fused to GFP (Inc-GFP) were grown in solid media under inducing (galactose; +GAL) or non-inducing (fructose; +FRU) conditions. After 48h, the Vps phenotype was analyzed qualitatively in solid media. Inc-GFP protein interfering with trafficking: CT22991-215-GFP; Negative control: GFP; Positive controls: the Legionella pneumophila effector VipA and the dominant-negative form of the yeast ATPase Vps4 (Vps4E233Q). Vps results with all yeast strains producing Inc-GFP proteins are summarized in S2 Table. (PDF) [file pone.0264292.s003.pdf]

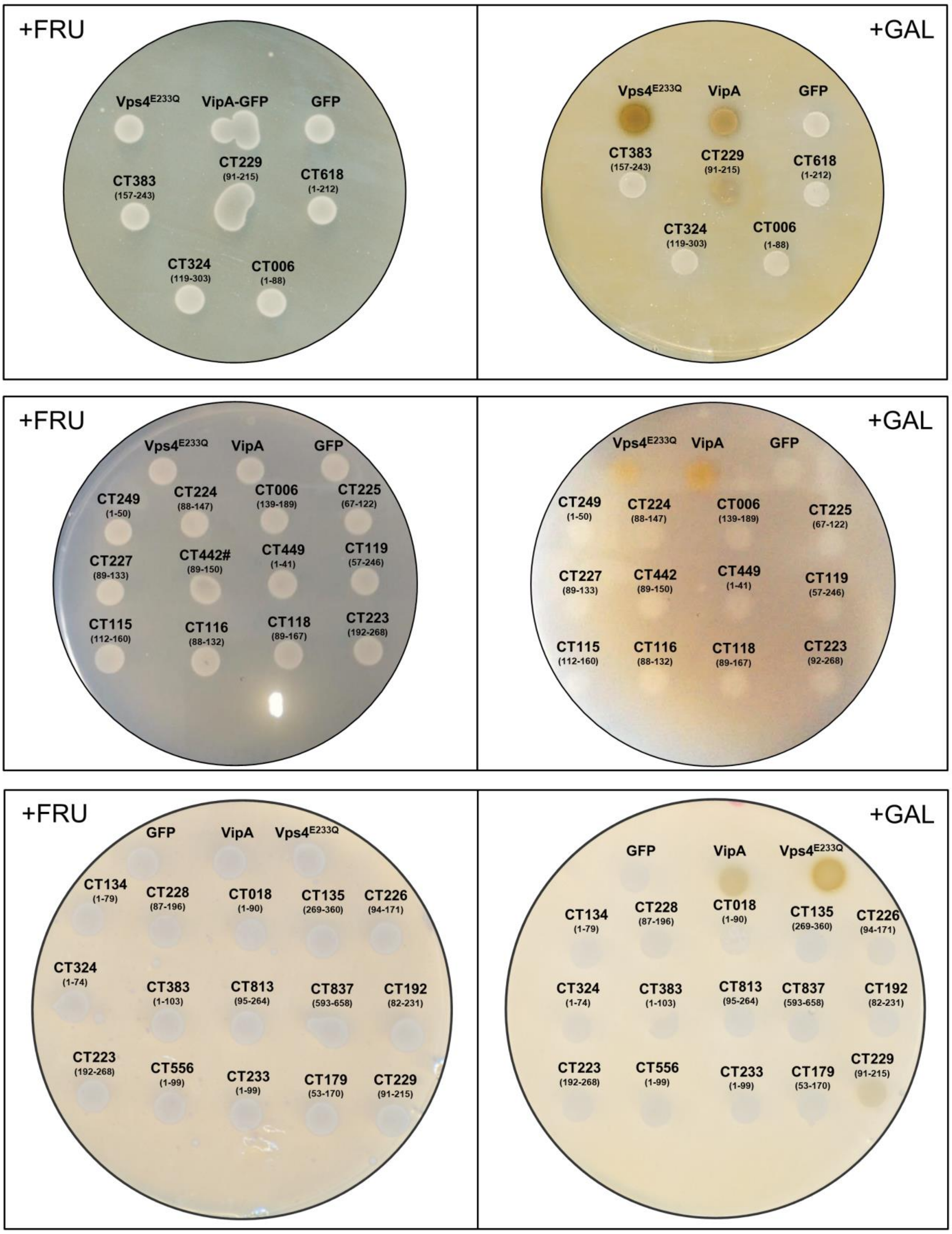

S3 Fig. The effect of Inc-GFP proteins on vacuolar protein sorting in yeast. *S. cerevisiae* NSY01 strains producing the indicated Inc fragments fused to GFP (Inc-GFP) were grown in solid media under inducing (galactose; +GAL) or non-inducing (fructose; +FRU) conditions. After 48 h, the Vps phenotype was analyzed qualitatively in solid media. Inc-GFP protein interfering with trafficking: CT229$_{91\text{-}215}$-GFP; Negative control: GFP; Positive controls: the *Legionella pneumophila* effector VipA and the dominant-negative form of the yeast ATPase Vps4 (Vps4^{E233Q}). Vps results with all yeast strains producing Inc-GFP proteins are summarized in S2 Table.
