# Supplementary material for: The Chlamydia trachomatis inclusion membrane protein CT006 associates with lipid droplets in eukaryotic cells
Source: PLoS One. 2022 Feb 22;17(2):e0264292. doi: 10.1371/journal.pone.0264292 (PMC8863265; doi:10.1371/journal.pone.0264292)
Supplement: S4 Fig — S. cerevisiae strains producing the indicated Inc fragments fused to GFP-Pep12L-TM (Inc-GFP-Pep12L-TM) were grown in solid media under inducing (galactose; +GAL) or non-inducing (fructose; +FRU) conditions. After 48 h, the Vps phenotype was analyzed qualitatively in solid media. Inc-GFP-Pep12L-TM protein interfering with trafficking: CT223192-268-GFP-Pep12L-TM; Negative controls: GFP and GFP-Pep12L-TM; Positive controls: the Legionella pneumophila effector VipA and the dominant-negative form of the yeast ATPase Vps4 (Vps4E233Q). *CT1351-209 is fused only to GFP (*CT1351-209-GFP). Vps results with all yeast strains producing Inc-GFP-Pep12L-TM proteins are summarized in S3 Table. (PDF) [file pone.0264292.s004.pdf]

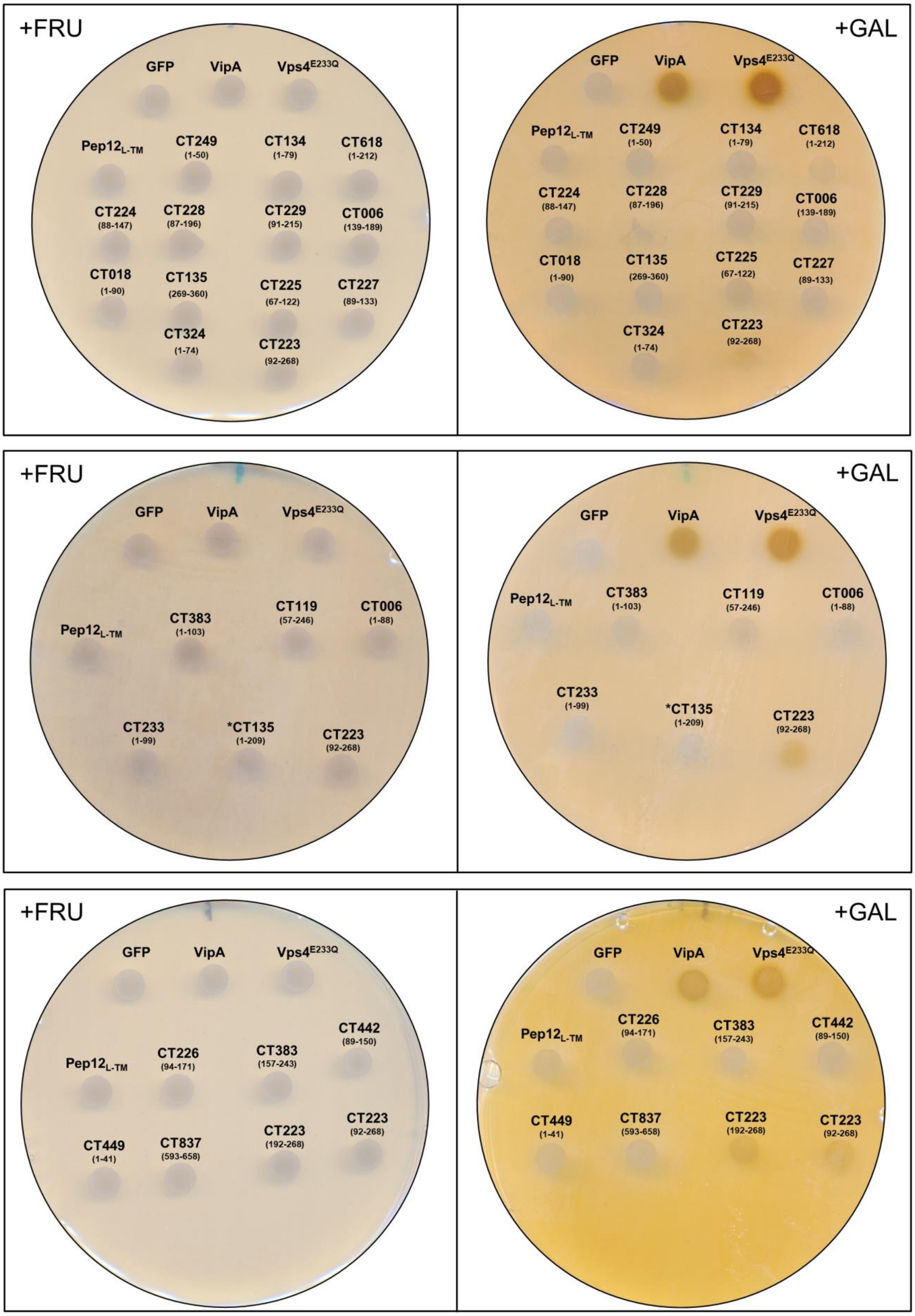

S4 Fig. The effect of Inc-GFP-Pep12$_{L\text{-}TM}$ proteins on vacuolar protein sorting in yeast. *S. cerevisiae* strains producing the indicated Inc fragments fused to GFP-Pep12$_{L\text{-}TM}$ (Inc-GFP-Pep12$_{L\text{-}TM}$) were grown in solid media under inducing (galactose; +GAL) or non-inducing (fructose; +FRU) conditions. After 48 h, the Vps phenotype was analyzed qualitatively in solid media. Inc-GFP-Pep12$_{L\text{-}TM}$ protein interfering with trafficking: CT223$_{192\text{-}268}$-GFP-Pep12$_{L\text{-}TM}$; Negative controls: GFP and GFP-Pep12$_{L\text{-}TM}$; Positive controls: the

Legionella pneumophila effector VipA and the dominant-negative form of the yeast ATPase Vps4 ($Vps4^{E233Q}$). *$CT135_{1\text{-}209}$ is fused only to GFP (*$CT135_{1\text{-}209}$-GFP). Vps results with all yeast strains producing Inc-GFP-$Pep12_{L\text{-}TM}$ proteins are summarized in S3 Table.
