# Supplementary material for: The Chlamydia trachomatis inclusion membrane protein CT006 associates with lipid droplets in eukaryotic cells
Source: PLoS One. 2022 Feb 22;17(2):e0264292. doi: 10.1371/journal.pone.0264292 (PMC8863265; doi:10.1371/journal.pone.0264292)
Supplement: S5 Fig — S. cerevisiae strains producing the indicated Inc-GFP proteins were grown in the presence of galactose. Live cells were visualized by fluorescence microscopy. Scale bars, 5 μm. (a) Examples for the intracellular localization of Inc-GFP proteins in yeast: cytosolic distribution (GFP and CT006139-189-GFP), mitochondria-like puncta (CT0181-90-GFP), endosomal compartments (CT22991-215-GFP) and lipid droplets (CT0061-88-GFP). (b) Examples for the intracellular localization of Inc-GFP-Pep12L-TM proteins at endosomal compartments in yeast. The intracellular localization of all Inc-GFP and Inc-GFP-Pep12L-TM fusion proteins analyzed in this study is summarized in S2 and S3 Tables. (PDF) [file pone.0264292.s005.pdf]

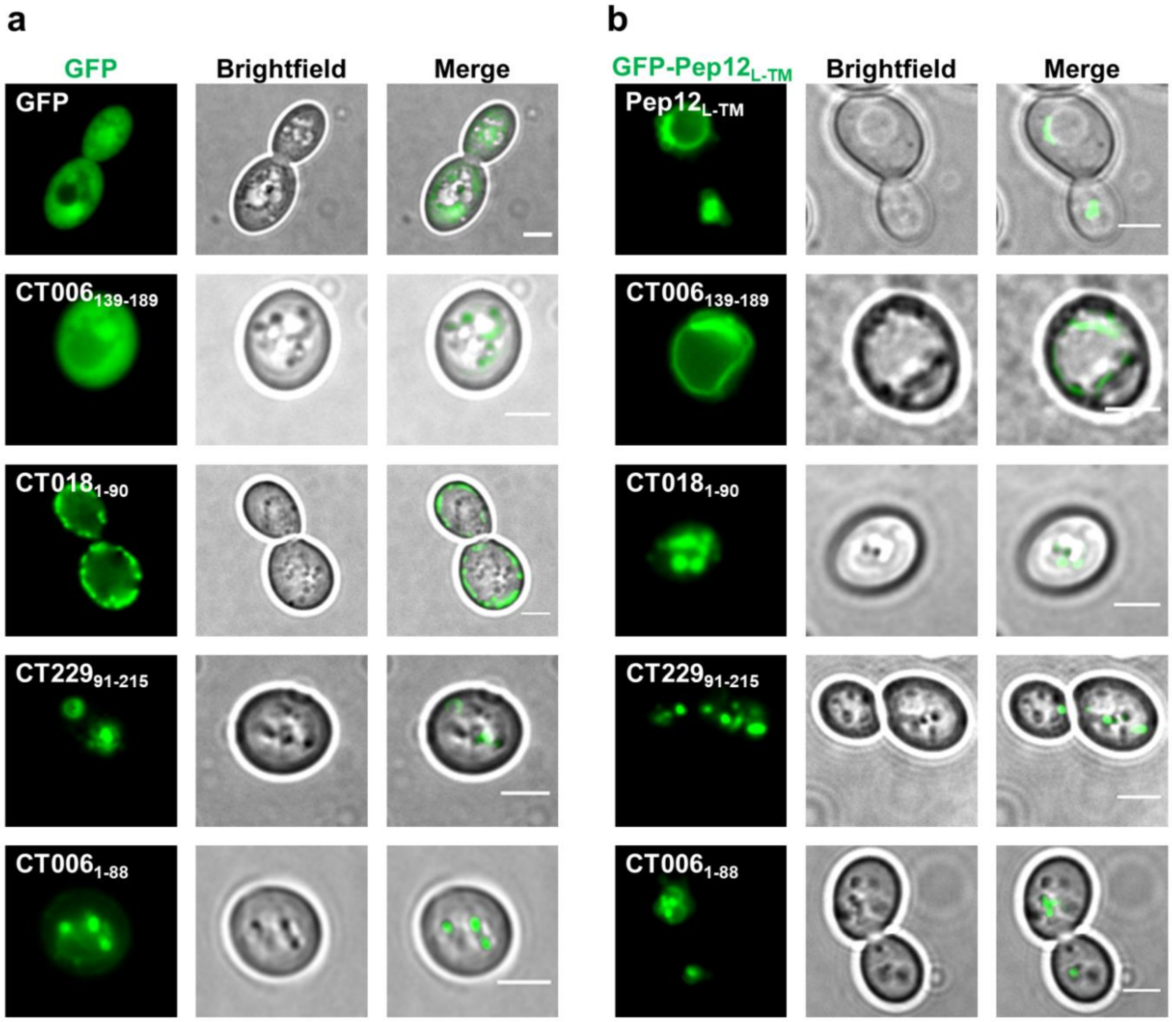


**S5 Fig. Intracellular localization of Inc-GFP and Inc-GFP-Pep12$_{L\text{-}TM}$ proteins in yeast.** *S. cerevisiae* strains producing the indicated Inc-GFP proteins were grown in the presence of galactose. Live cells were visualized by fluorescence microscopy. Scale bars, 5 µm. (a) Examples for the intracellular localization of Inc-GFP proteins in yeast: cytosolic distribution (GFP and CT006$_{139\text{-}189}$-GFP), mitochondria-like puncta (CT018$_{1\text{-}90}$-GFP), endosomal compartments (CT229$_{91\text{-}215}$-GFP) and lipid droplets (CT006$_{1\text{-}88}$-GFP). (b) Examples for the intracellular localization of Inc-GFP-Pep12$_{L\text{-}TM}$ proteins at endosomal compartments in yeast. The intracellular localization of all Inc-GFP and Inc-GFP-Pep12$_{L\text{-}TM}$ fusion proteins analyzed in this study is summarized in S2 and S3 Tables.
