# Supplementary material for: The Chlamydia trachomatis inclusion membrane protein CT006 associates with lipid droplets in eukaryotic cells
Source: PLoS One. 2022 Feb 22;17(2):e0264292. doi: 10.1371/journal.pone.0264292 (PMC8863265; doi:10.1371/journal.pone.0264292)
Supplement: S6 Fig — According to TMHMM server, v 2.0 (Sonnhammer ELL, Krogh A. A hidden Markov model for predicting transmembrane helices in protein sequence. Sixth Int Conf Intell Syst Mol Biol. 1998;6:175–182; Krogh A, Larsson B, Von Heijne G, Sonnhammer ELL. Predicting transmembrane protein topology with a hidden Markov model: Application to complete genomes. J Mol Biol. 2001;305(3):567–580). CT006 is predicted to have 3 transmembrane domains between amino acid residues 47 and 69, 89 and 111, and 118 and 140. The positions of the last two hydrophobic motifs are approximately in agreement with the prediction of the bilobed hydrophobic motif described by Dehoux et al, 2011 (Dehoux P, Flores R, Dauga C, Zhong G, Subtil A. Multi-genome identification and characterization of chlamydiae-specific type III secretion substrates: The Inc proteins. BMC Genomics. 2011;12:109). (PDF) [file pone.0264292.s006.pdf]

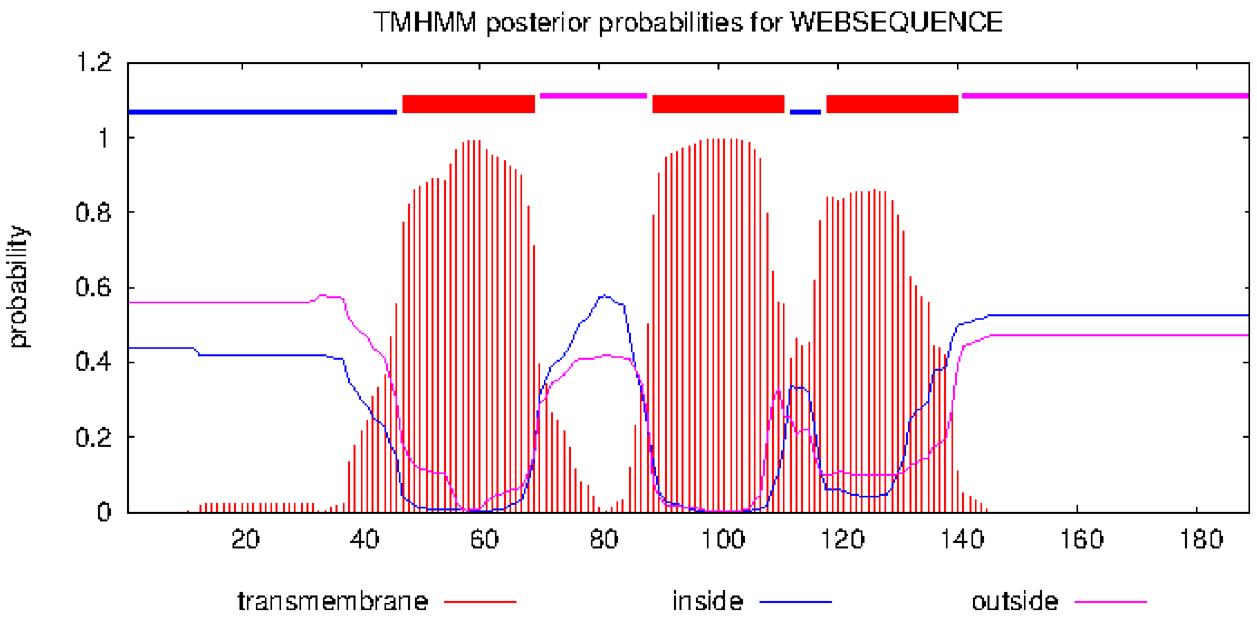

S6 Fig. Prediction of transmembrane helices in CT006. According to TMHMM server, v 2.0 (Sonnhammer ELL, Krogh A. A hidden Markov model for predicting transmembrane helices in protein sequence. Sixth Int Conf Intell Syst Mol Biol. 1998;6:175–182; Krogh A, Larsson B, Von Heijne G, Sonnhammer ELL. Predicting transmembrane protein topology with a hidden Markov model: Application to complete genomes. J Mol Biol. 2001;305(3):567–580). CT006 is predicted to have 3 transmembrane domains between amino acid residues 47 and 69, 89 and 111, and 118 and 140. The positions of the last two hydrophobic motifs are approximately in agreement with the prediction of the bilobed hydrophobic motif described by Dehoux *et al*, 2011 (Dehoux P, Flores R, Dauga C, Zhong G, Subtil A. Multi-genome identification and characterization of chlamydiae-specific type III secretion substrates: The Inc proteins. BMC Genomics. 2011;12:109).
