# Supplementary material for: The Chlamydia trachomatis inclusion membrane protein CT006 associates with lipid droplets in eukaryotic cells
Source: PLoS One. 2022 Feb 22;17(2):e0264292. doi: 10.1371/journal.pone.0264292 (PMC8863265; doi:10.1371/journal.pone.0264292)
Supplement: S7 Fig — HeLa 229 cells were transfected for 24 h with plasmids encoding mEGFP or the indicated versions of CT006 containing a mEGFP tag at their amino-termini (mEGFP-CT006 proteins) or at their carboxy-termini (CT006-mEGFP proteins). (a) Transfected cells were fixed with 4% (w/v) PFA and imaged by fluorescence microscopy. Scale bars, 10 μm. (b) Whole cell extracts were analyzed by immunoblotting with antibodies against GFP and α-tubulin (HeLa 229 cells loading control) and appropriate HRP-conjugated secondary antibodies. Proteins were detected using SuperSignal West Pico detection kit (Thermo Fisher Scientific). The crosses in (a) correspond to proteins that were not analyzed in this study. (PDF) [file pone.0264292.s007.pdf]

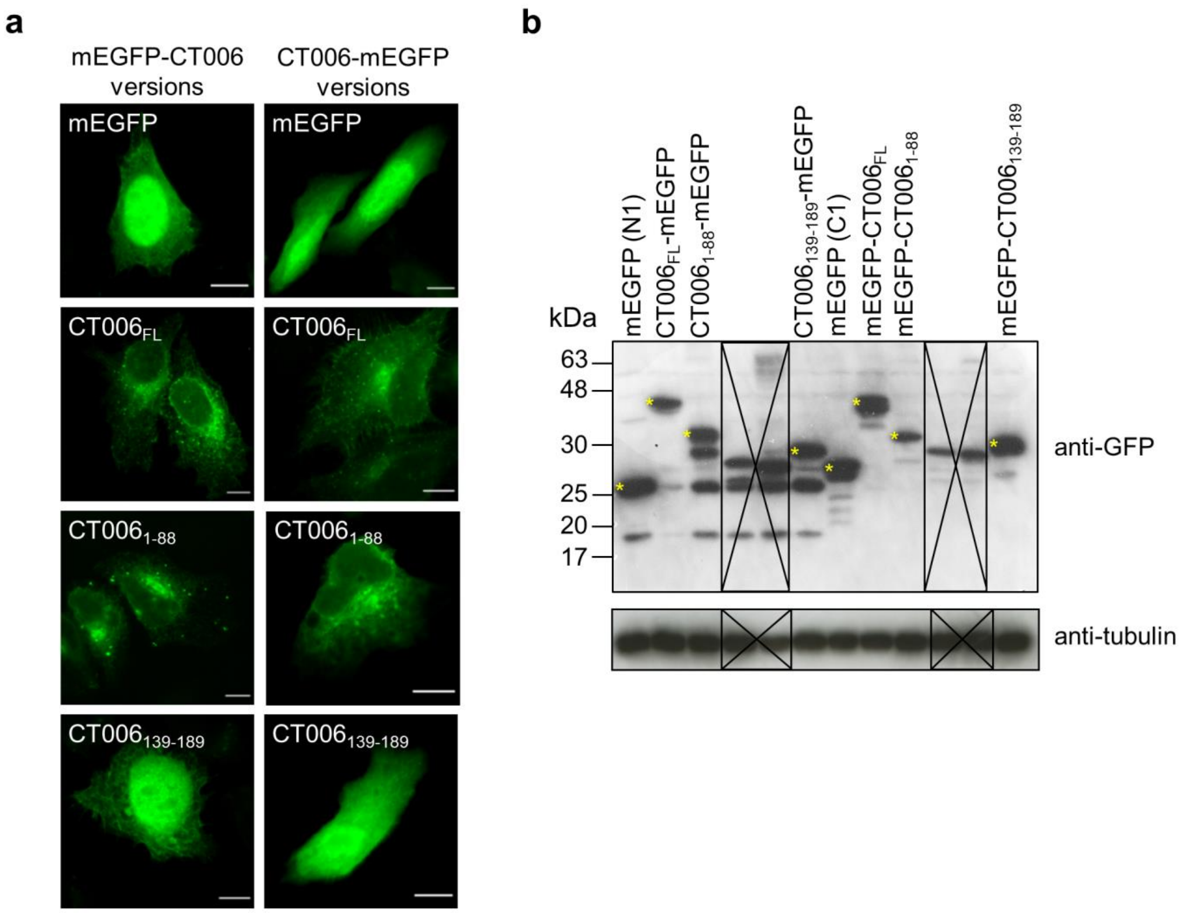

S7 Fig. Analysis of the production and intracellular localization of CT006 versions in mammalian cells. HeLa 229 cells were transfected for 24 h with plasmids encoding mEGFP or the indicated versions of CT006 containing a mEGFP tag at their amino-termini (mEGFP-CT006 proteins) or at their carboxy-termini (CT006-mEGFP proteins). (a) Transfected cells were fixed with 4% (w/v) PFA and imaged by fluorescence microscopy. Scale bars, 10 µm. (b) Whole cell extracts were analyzed by immunoblotting with antibodies against GFP and α-tubulin (HeLa 229 cells loading control) and appropriate HRP-conjugated secondary antibodies. Proteins were detected using SuperSignal West Pico detection kit (Thermo Fisher Scientific). The crosses in (a) correspond to proteins that were not analyzed in this study.
