# Supplementary material for: The Chlamydia trachomatis inclusion membrane protein CT006 associates with lipid droplets in eukaryotic cells
Source: PLoS One. 2022 Feb 22;17(2):e0264292. doi: 10.1371/journal.pone.0264292 (PMC8863265; doi:10.1371/journal.pone.0264292)
Supplement: S8 Fig — HeLa 229 cells were transfected with plasmids encoding mEGFP or the indicated versions of CT006 containing a mEGFP tag at their amino-termini (mEGFP-CT006 proteins). After 18 h, cells were treated ethanol (solvent control; left-hand side panel) or 100 μM oleic acid (right-hand side panel) for 6 h and fixed with 4% (w/v) PFA. Fixed cells were immunolabeled with an antibody against Protein disulfide isomerase (PDI), and an appropriate fluorophore-conjugated secondary antibody, and imaged by fluorescence microscopy. Scale bars, 10 μm. (PDF) [file pone.0264292.s008.pdf]

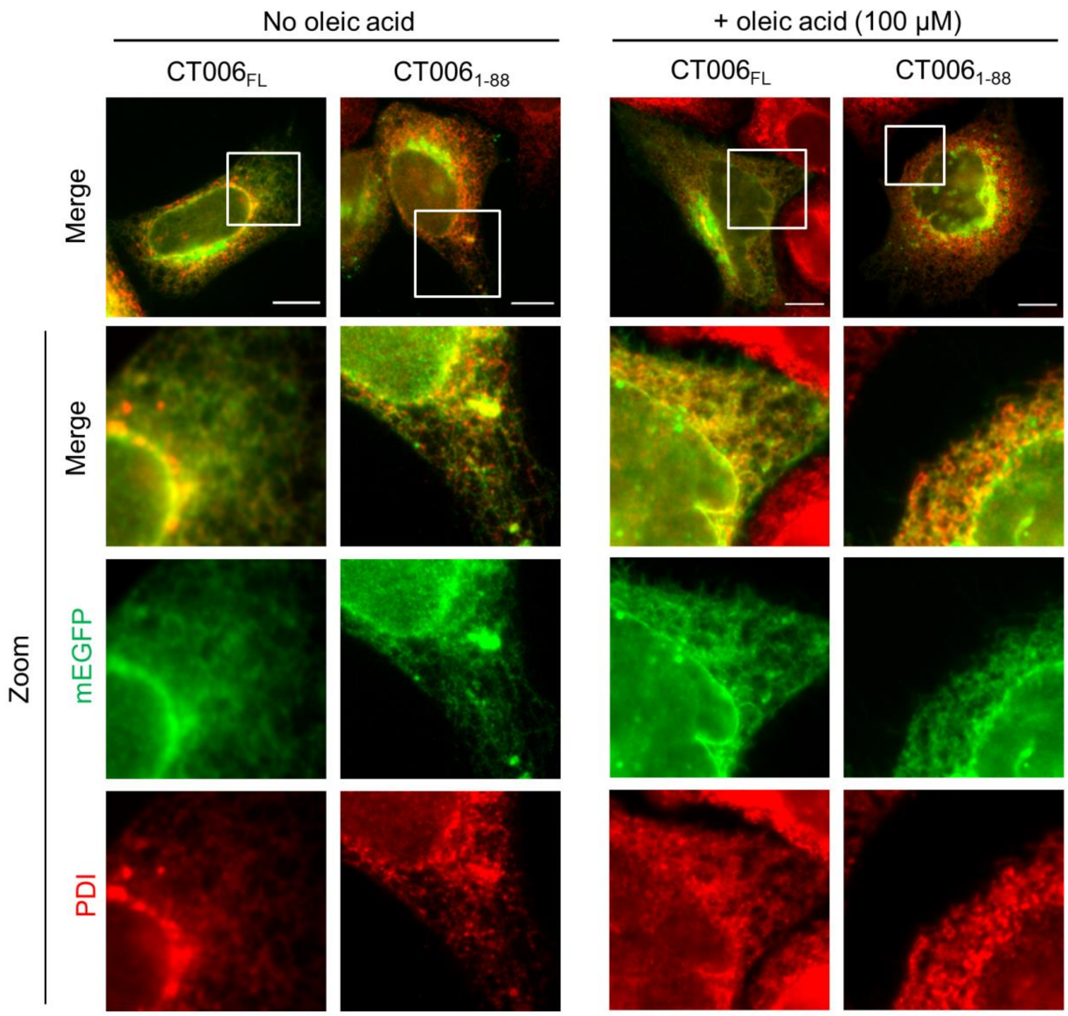

S8 Fig. Full-length CT006 and CT006$_{1-88}$ fused to mEGFP partially co-localize with the endoplasmic reticulum in mammalian cells. HeLa 229 cells were transfected with plasmids encoding mEGFP or the indicated versions of CT006 containing a mEGFP tag at their amino-termini (mEGFP-CT006 proteins). After 18 h, cells were treated ethanol (solvent control; left-hand side panel) or 100 μM oleic acid (right-hand side panel) for 6 h and fixed with 4% (w/v) PFA. Fixed cells were immunolabeled with an antibody against Protein disulfide isomerase (PDI), and an appropriate fluorophore-conjugated secondary antibody, and imaged by fluorescence microscopy. Scale bars, 10 μm.
