# Supplementary material for: The Chlamydia trachomatis inclusion membrane protein CT006 associates with lipid droplets in eukaryotic cells
Source: PLoS One. 2022 Feb 22;17(2):e0264292. doi: 10.1371/journal.pone.0264292 (PMC8863265; doi:10.1371/journal.pone.0264292)
Supplement: S9 Fig — HeLa 229 cells were transfected for 24 h with plasmids encoding mEGFP or the indicated versions of CT0061-88 containing a mEGFP tag at their amino-termini. Whole cell extracts were analyzed by immunoblotting with antibodies against GFP and α-tubulin (HeLa 229 cells loading control) and appropriate HRP-conjugated secondary antibodies. Proteins were detected using SuperSignal West Pico detection kit (Thermo Fisher Scientific). (PDF) [file pone.0264292.s009.pdf]

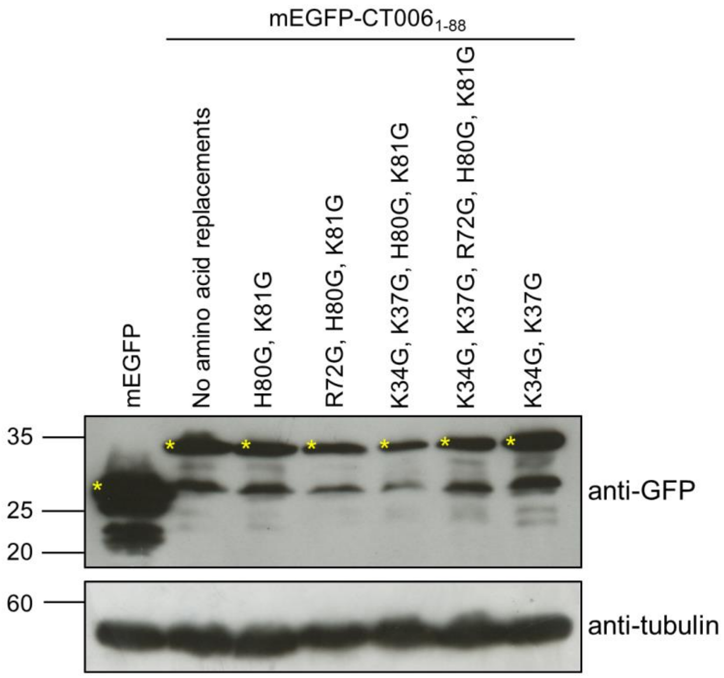

S9 Fig. Analysis of the production of mEGFP-CT006$_{1-88}$ versions with positively charged amino acids replaced by glycines in mammalian HeLa cells. HeLa 229 cells were transfected for 24 h with plasmids encoding mEGFP or the indicated versions of CT006$_{1-88}$ containing a mEGFP tag at their amino-termini. Whole cell extracts were analyzed by immunoblotting with antibodies against GFP and α-tubulin (HeLa 229 cells loading control) and appropriate HRP-conjugated secondary antibodies. Proteins were detected using SuperSignal West Pico detection kit (Thermo Fisher Scientific).
