# Supplementary material for: The Chlamydia trachomatis inclusion membrane protein CT006 associates with lipid droplets in eukaryotic cells
Source: PLoS One. 2022 Feb 22;17(2):e0264292. doi: 10.1371/journal.pone.0264292 (PMC8863265; doi:10.1371/journal.pone.0264292)
Supplement: S10 Fig — HeLa 229 cells were infected by C. trachomatis L2/434 or C. trachomatis L2/434 harboring pCT006-2HA (L2/434+pCT006-2HA). At the indicated hours post-infection (hpi), cells were fixed with 4% (w/v) PFA, immunolabeled with antibodies against HA (red), C. trachomatis Hsp60 (green), and appropriate fluorophore-conjugated secondary antibodies, and imaged by fluorescence microscopy. Scale bars, 10 μm. Dashed lines represent the limits of infected HeLa cells and the chlamydial inclusions within were zoomed at 2, 4, 6 and 8 hpi. (PDF) [file pone.0264292.s010.pdf]

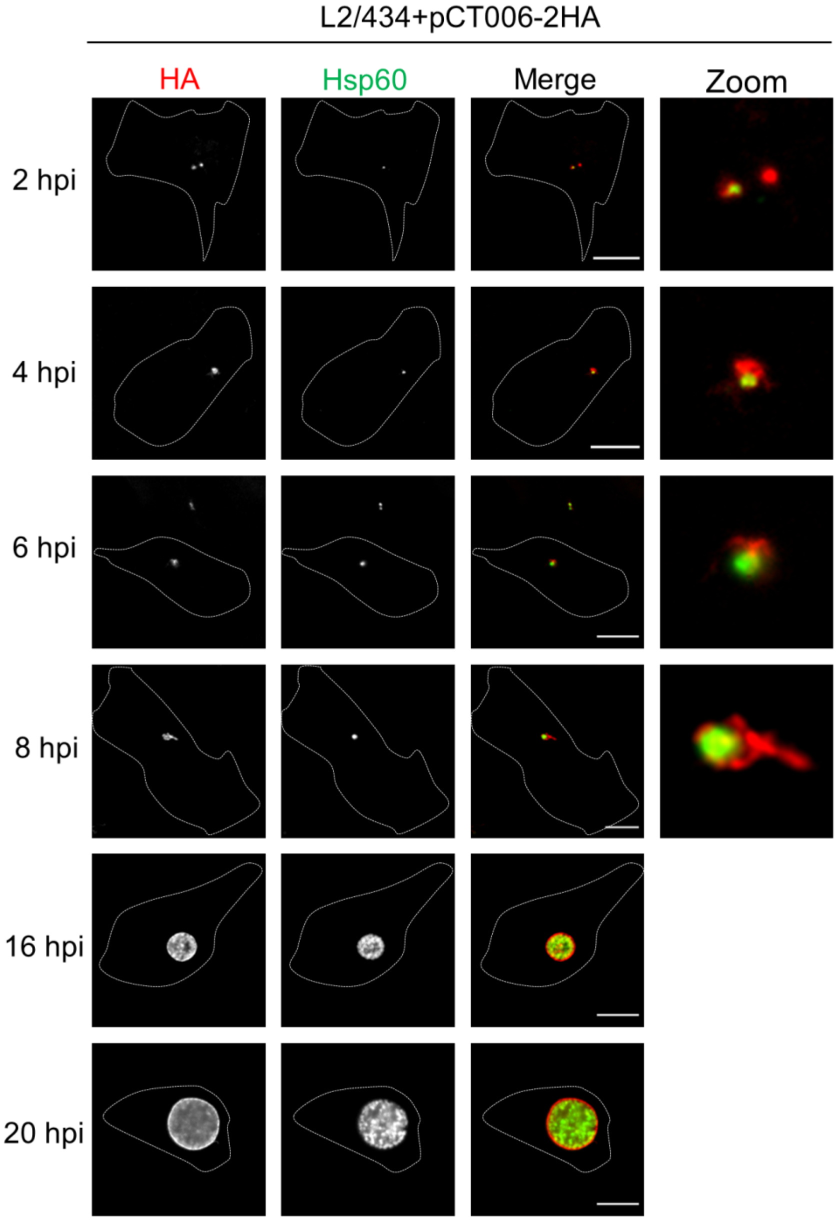

S10 Fig. Plasmid-encoded CT006-2HA is produced at early times post-infection and accumulates at the periphery of the inclusion. HeLa 229 cells were infected by *C. trachomatis* L2/434 or *C. trachomatis* L2/434 harboring pCT006-2HA (L2/434+pCT006-2HA). At the indicated hours post-infection (hpi), cells were fixed with 4% (w/v) PFA, immunolabeled with antibodies against HA (red), *C. trachomatis* Hsp60 (green), and appropriate fluorophore-conjugated secondary antibodies, and imaged by fluorescence microscopy. Scale bars, 10 µm. Dashed lines represent the limits of infected HeLa cells and the chlamydial inclusions within were zoomed at 2, 4, 6 and 8 hpi.
