# Supplementary material for: The Chlamydia trachomatis inclusion membrane protein CT006 associates with lipid droplets in eukaryotic cells
Source: PLoS One. 2022 Feb 22;17(2):e0264292. doi: 10.1371/journal.pone.0264292 (PMC8863265; doi:10.1371/journal.pone.0264292)
Supplement: S11 Fig — HeLa 229 cells were transfected with plasmids encoding mEGFP or different versions of CT449 containing a mEGFP tag at their amino-termini (mEGFP-CT449FL, mEGFP-CT4491-41 or mEGFP-CT44988-110). (a) At 24 h post-transfection, whole cell extracts were analyzed by immunoblotting with antibodies against GFP and α-tubulin (HeLa 229 cells loading control) and appropriate HRP-conjugated secondary antibodies. (b) At 24 h post-transfection, cells were fixed with 4% (w/v) PFA and analyzed by fluorescence microscopy. (c) At 18 h post-transfection, cells were treated with 100 μM oleic acid for 6 h and then fixed with 4% (w/v) PFA. Fixed cells were labeled with anti-GFP and the appropriate fluorophore-conjugated secondary antibody, stained with Oil Red O (3:2 v/v Oil Red O stock solution diluted in water), and imaged by fluorescence microscopy. Scale bars, 10 μm. The area delimited by a white square was zoomed. (PDF) [file pone.0264292.s011.pdf]

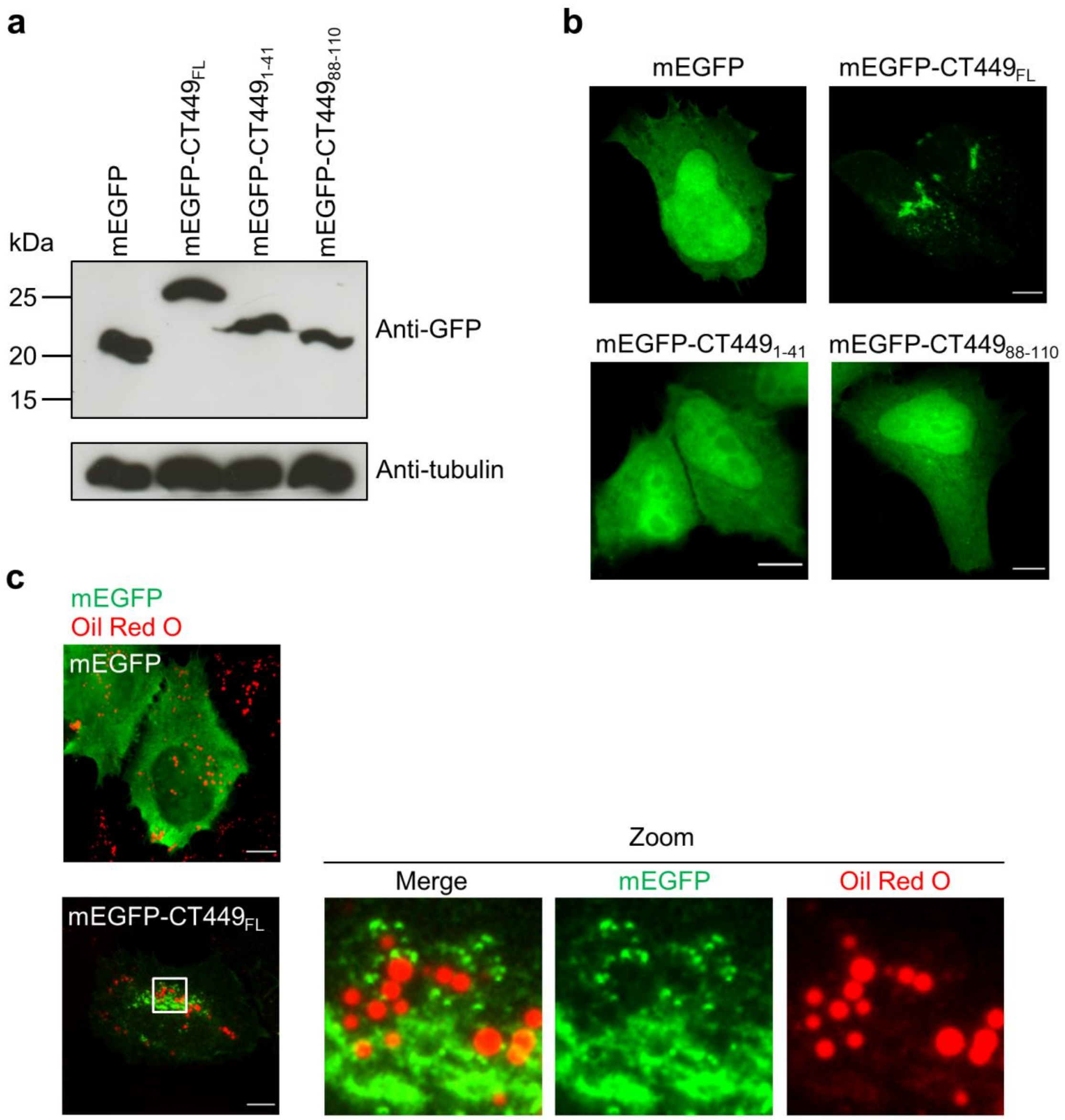

S11 Fig. mEGFP-CT449 does not localize at lipid droplets in mammalian cells. HeLa 229 cells were transfected with plasmids encoding mEGFP or different versions of CT449 containing a mEGFP tag at their amino-termini (mEGFP-CT449$_{FL}$, mEGFP-CT449$_{1-41}$ or mEGFP-CT449$_{88-110}$). (a) At 24 h post-transfection, whole cell extracts were analyzed by immunoblotting with antibodies against GFP and α-tubulin (HeLa 229 cells loading control) and appropriate HRP-conjugated secondary antibodies. (b) At 24 h post-transfection, cells were fixed with 4% (w/v) PFA and analyzed by fluorescence microscopy. (c) At 18 h post-transfection, cells were treated with 100 μM oleic acid for 6 h and then fixed with 4% (w/v) PFA. Fixed cells were labeled with anti-GFP and the appropriate fluorophore-conjugated secondary antibody, stained with Oil Red O (3:2 v/v Oil Red O stock solution diluted in water), and imaged by fluorescence microscopy. Scale bars, 10 μm. The area delimited by a white square was zoomed.
