# Supplementary material for: The Chlamydia trachomatis inclusion membrane protein CT006 associates with lipid droplets in eukaryotic cells
Source: PLoS One. 2022 Feb 22;17(2):e0264292. doi: 10.1371/journal.pone.0264292 (PMC8863265; doi:10.1371/journal.pone.0264292)
Supplement: S12 Fig — HeLa 229 cells were left uninfected (UI) or infected by C. trachomatis L2/434 or by L2/434 strains harboring (a) pCT449-2HA, (b) pCT0065G-2HA, or (c) pCT006Δ47-67-2HA. At the indicated times post-infection, whole cell extracts were analyzed by immunoblotting using antibodies against HA, Hsp60 (bacterial loading control) and α-tubulin (HeLa 229 cells loading control) and the appropriate HRP-conjugated secondary antibodies, followed by detection using SuperSignal West Pico detection kit (Thermo Fisher Scientific). (PDF) [file pone.0264292.s012.pdf]

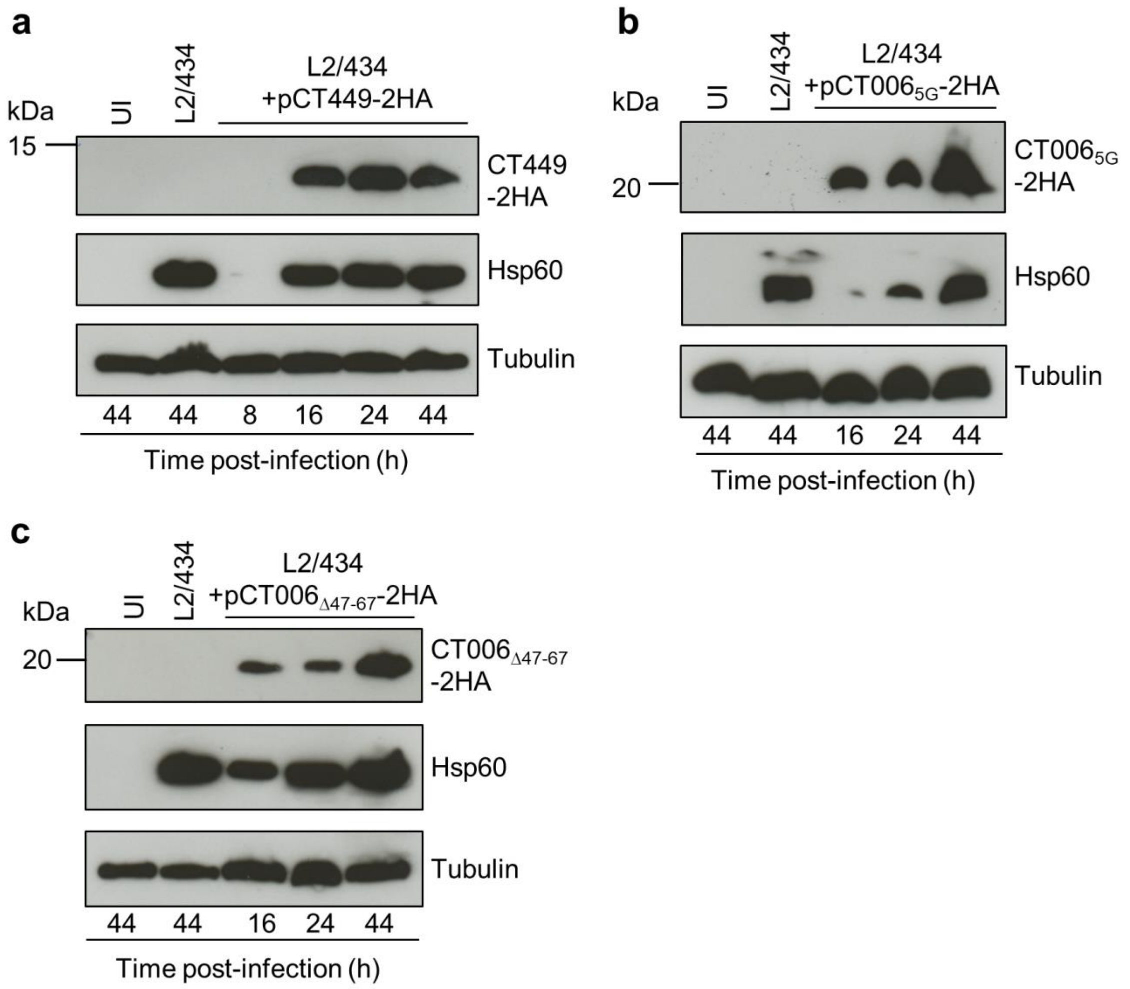

S12 Fig. Analysis of the production of plasmid-encoded CT449-2HA, CT006$_{5G}$-2HA and CT006$_{\Delta 47-67}$-2HA by *C. trachomatis*. HeLa 229 cells were left uninfected (UI) or infected by *C. trachomatis* L2/434 or by L2/434 strains harboring (a) pCT449-2HA, (b) pCT006$_{5G}$-2HA, or (c) pCT006$_{\Delta 47-67}$-2HA. At the indicated times post-infection, whole cell extracts were analyzed by immunoblotting using antibodies against HA, Hsp60 (bacterial loading control) and α-tubulin (HeLa 229 cells loading control) and the appropriate HRP-conjugated secondary antibodies, followed by detection using SuperSignal West Pico detection kit (Thermo Fisher Scientific).
