# Supplementary material for: The Chlamydia trachomatis inclusion membrane protein CT006 associates with lipid droplets in eukaryotic cells
Source: PLoS One. 2022 Feb 22;17(2):e0264292. doi: 10.1371/journal.pone.0264292 (PMC8863265; doi:10.1371/journal.pone.0264292)
Supplement: S13 Fig — HeLa 229 cells were infected by L2/434 strains harboring pCT006-2HA, pCT449-2HA, pCT0065G-2HA or pCT006Δ47-67-2HA. At the indicated times post-infection, infected cells were fixed with 4% (w/v) PFA, immunolabeled with antibodies against HA (red), Hsp60 (green) and appropriate fluorophore-conjugated secondary antibodies. The labeled cells were then imaged by fluorescence microscopy. Scale bars, 10 μm. (PDF) [file pone.0264292.s013.pdf]

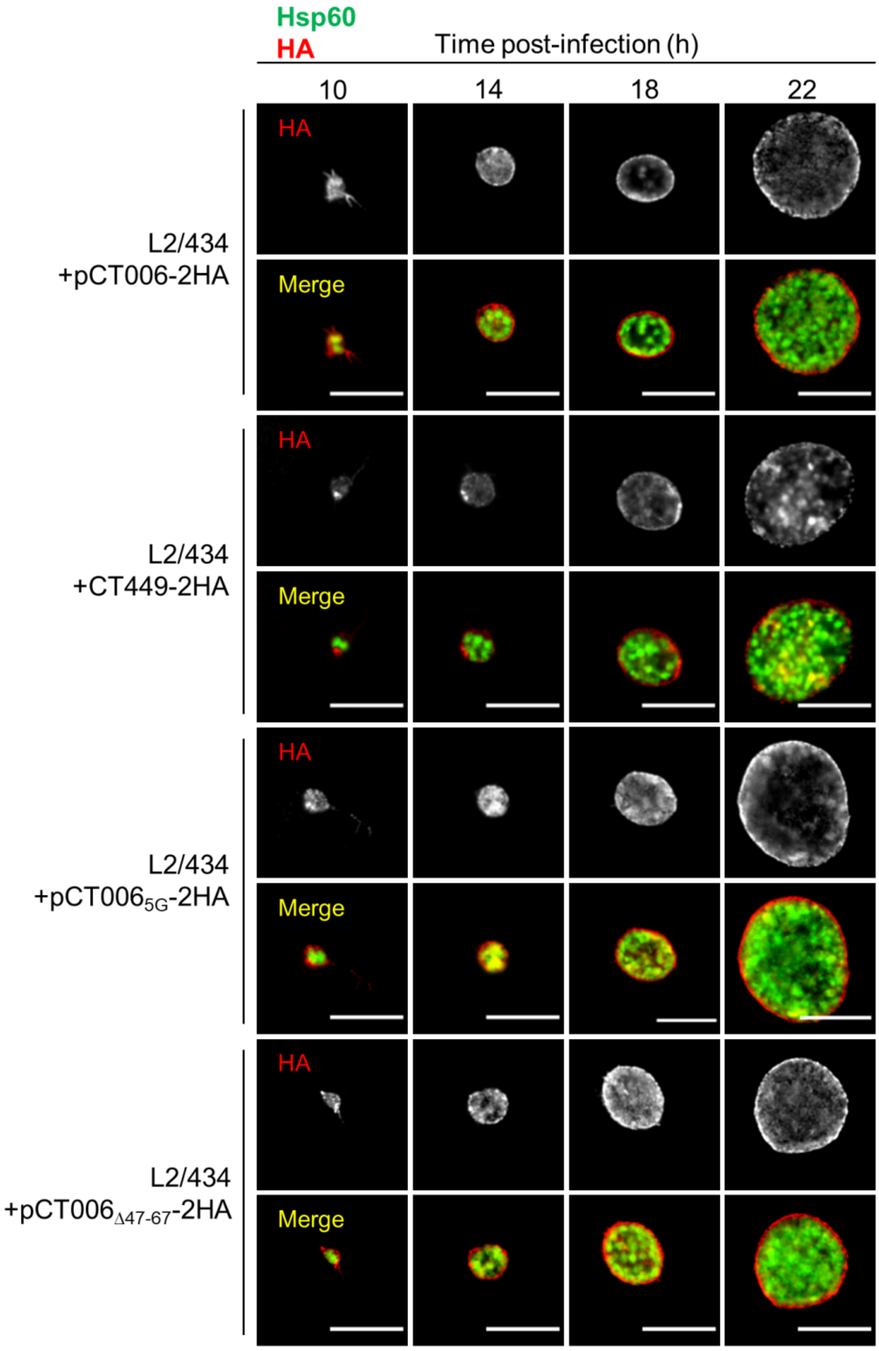

S13 Fig. Analysis of the intracellular localization of plasmid-encoded CT006-2HA, CT449-2HA, CT006$_{5G}$-2HA and CT006$_{\Delta 47\text{-}67}$-2HA. HeLa 229 cells were infected by L2/434 strains harboring pCT006-2HA, pCT449-2HA, pCT006$_{5G}$-2HA or pCT006$_{\Delta 47\text{-}67}$-2HA. At the indicated times post-infection, infected cells were fixed with 4% (w/v) PFA, immunolabeled with antibodies against HA (red), Hsp60 (green) and appropriate fluorophore-conjugated secondary antibodies. The labeled cells were then imaged by fluorescence microscopy. Scale bars, 10 μm.
