# Supplementary material for: The Chlamydia trachomatis inclusion membrane protein CT006 associates with lipid droplets in eukaryotic cells
Source: PLoS One. 2022 Feb 22;17(2):e0264292. doi: 10.1371/journal.pone.0264292 (PMC8863265; doi:10.1371/journal.pone.0264292)
Supplement: S14 Fig — HeLa 229 cells were infected by C. trachomatis L2/434 strains harboring pCT449-2HA, pCT0065G-2HA or pCT006Δ47-67-2HA. At the indicated times post-infection, infected cells previously treated with 100 μM oleic acid for 6 h were fixed with 4% (w/v) PFA, immunolabeled with an antibody against Hsp60 (red) and an appropriate fluorophore-conjugated secondary antibody, stained with the neutral lipid dye BODIPY (green) and imaged by fluorescence microscopy. Dashed lines represent the limits of infected HeLa 229 cells. Scale bars, 10 μm. (PDF) [file pone.0264292.s014.pdf]

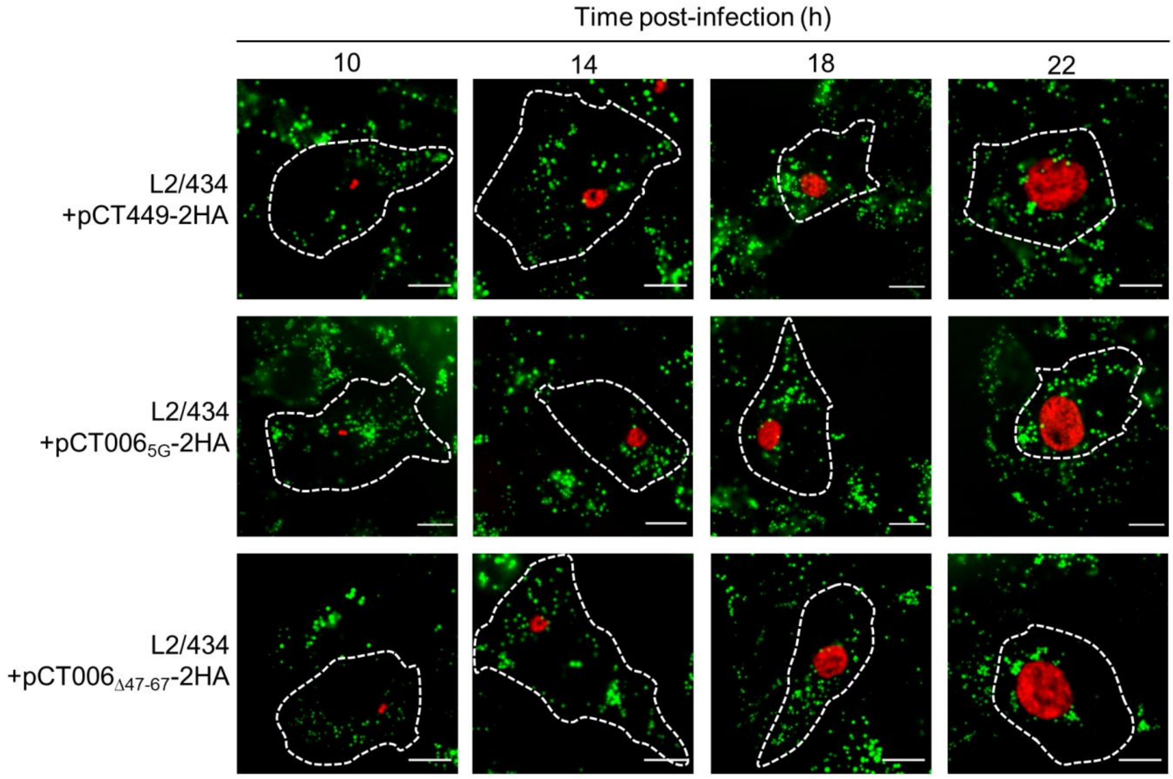

S14 Fig. Analysis of the localization of LDs in cells infected by *C. trachomatis*. HeLa 229 cells were infected by *C. trachomatis* L2/434 strains harboring pCT449-2HA, pCT006$_{5G}$-2HA or pCT006$_{\Delta47-67}$-2HA. At the indicated times post-infection, infected cells previously treated with 100 μM oleic acid for 6 h were fixed with 4% (w/v) PFA, immunolabeled with an antibody against Hsp60 (red) and an appropriate fluorophore-conjugated secondary antibody, stained with the neutral lipid dye BODIPY (green) and imaged by fluorescence microscopy. Dashed lines represent the limits of infected HeLa 229 cells. Scale bars, 10 μm.
