# Supplementary material for: The Chlamydia trachomatis inclusion membrane protein CT006 associates with lipid droplets in eukaryotic cells
Source: PLoS One. 2022 Feb 22;17(2):e0264292. doi: 10.1371/journal.pone.0264292 (PMC8863265; doi:10.1371/journal.pone.0264292)
Supplement: S15 Fig — (a) HeLa 229 cells were infected by the C. trachomatis L2/434 parental strain or by C. trachomatis L2/434 strains harboring pCT006-2HA (L2/434+pCT006-2HA) or pCT449-2HA (L2/434+pCT449-2HA). (b) HeLa 229 cells were infected by the C. trachomatis L2/434 parental strain or by C. trachomatis L2/434 strains harboring pCT006-2HA (L2/434+pCT006-2HA), pCT0065G-2HA (L2/434+pCT0065G-2HA) or pCT449-2HA (L2/434+pCT449-2HA). The area of BODIPY-positive LDs at the region of chlamydial inclusions was measured from randomly selected images, as those depicted in Fig 7a and S14 Fig, using Fiji software (Schindelin J, Arganda-Carreras I, Frise E, Kaynig V, Longair M, Pietzsch T, et al. Fiji: An open-source platform for biological-image analysis. Nat Methods. 2012;9(7):676–682). Data are represented as the mean and standard error of the mean from three independent experiments (n = 60). Statistical analysis was performed using Kruskal-Wallis test with Dunn’s multiple comparison test. * Represents P<0.05 by comparison to the L2/434 strain; ϕ represents P<0.05 by comparison to the L2/434+pCT006-2HA. (PDF) [file pone.0264292.s015.pdf]

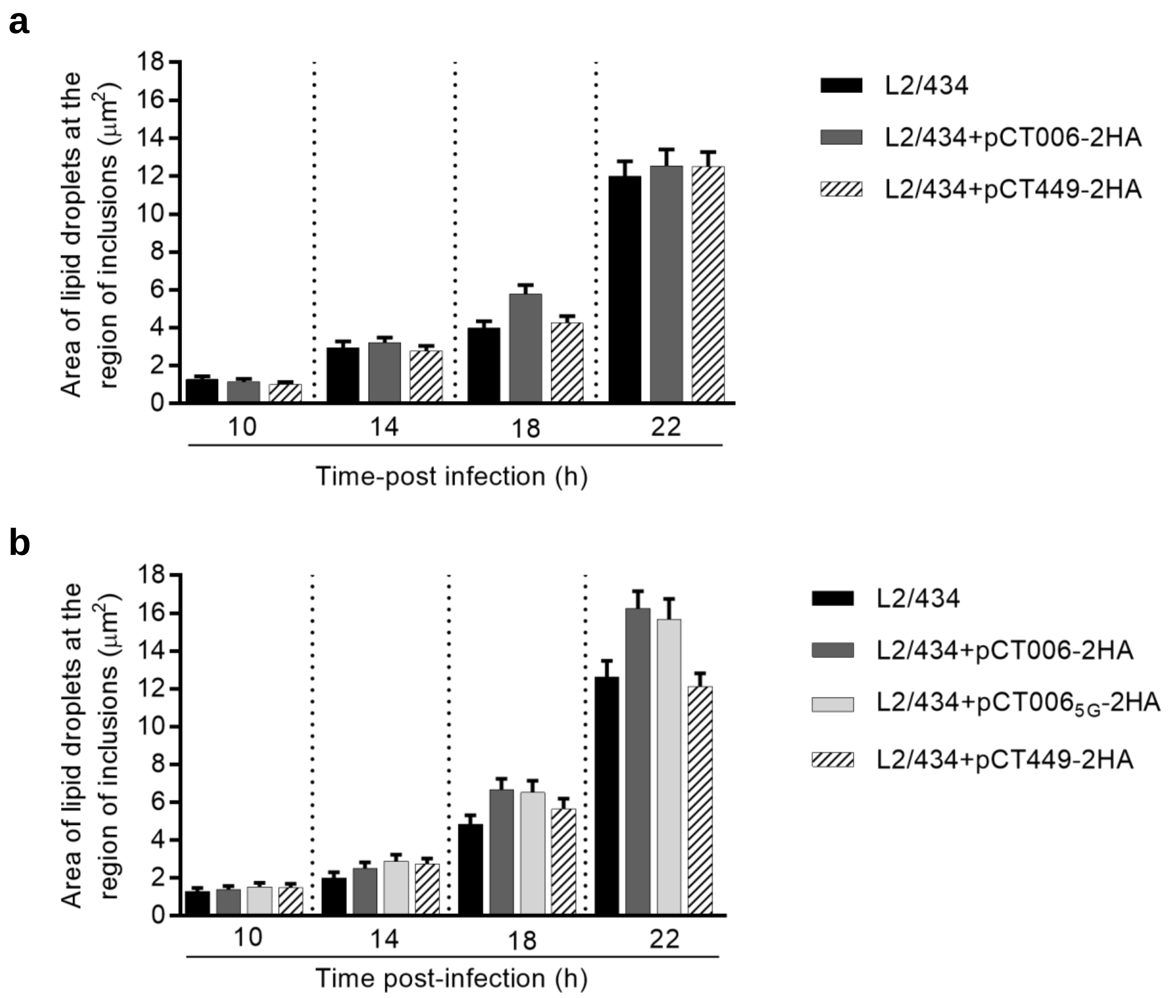

S15 Fig. Analysis of the area of LDs at the region of inclusions. (a) HeLa 229 cells were infected by the *C. trachomatis* L2/434 parental strain or by *C. trachomatis* L2/434 strains harboring pCT006-2HA (L2/434+pCT006-2HA) or pCT449-2HA (L2/434+pCT449-2HA). (b) HeLa 229 cells were infected by the *C. trachomatis* L2/434 parental strain or by *C. trachomatis* L2/434 strains harboring pCT006-2HA (L2/434+pCT006-2HA), pCT006$_{5G}$-2HA (L2/434+pCT006$_{5G}$-2HA) or pCT449-2HA (L2/434+pCT449-2HA). The area of BODIPY-positive LDs at the region of chlamydial inclusions was measured from randomly selected images, as those depicted in Figs 7a and S14, using Fiji software (Schindelin J, Arganda-Carreras I, Frise E, Kaynig V, Longair M, Pietzsch T, *et al.* Fiji: An open-source platform for biological-image analysis. Nat Methods. 2012;9(7):676–682). Data are represented as the mean and standard error of the mean from three independent experiments (n=60). Statistical analysis was performed using Kruskal-Wallis test with Dunn's multiple comparison test. * Represents P<0.05 by comparison to the L2/434 strain; ϕ represents P<0.05 by comparison to the L2/434+pCT006-2HA.
