# Supplementary material for: The Chlamydia trachomatis inclusion membrane protein CT006 associates with lipid droplets in eukaryotic cells
Source: PLoS One. 2022 Feb 22;17(2):e0264292. doi: 10.1371/journal.pone.0264292 (PMC8863265; doi:10.1371/journal.pone.0264292)

1. Uncropped blot images from Fig 5a.

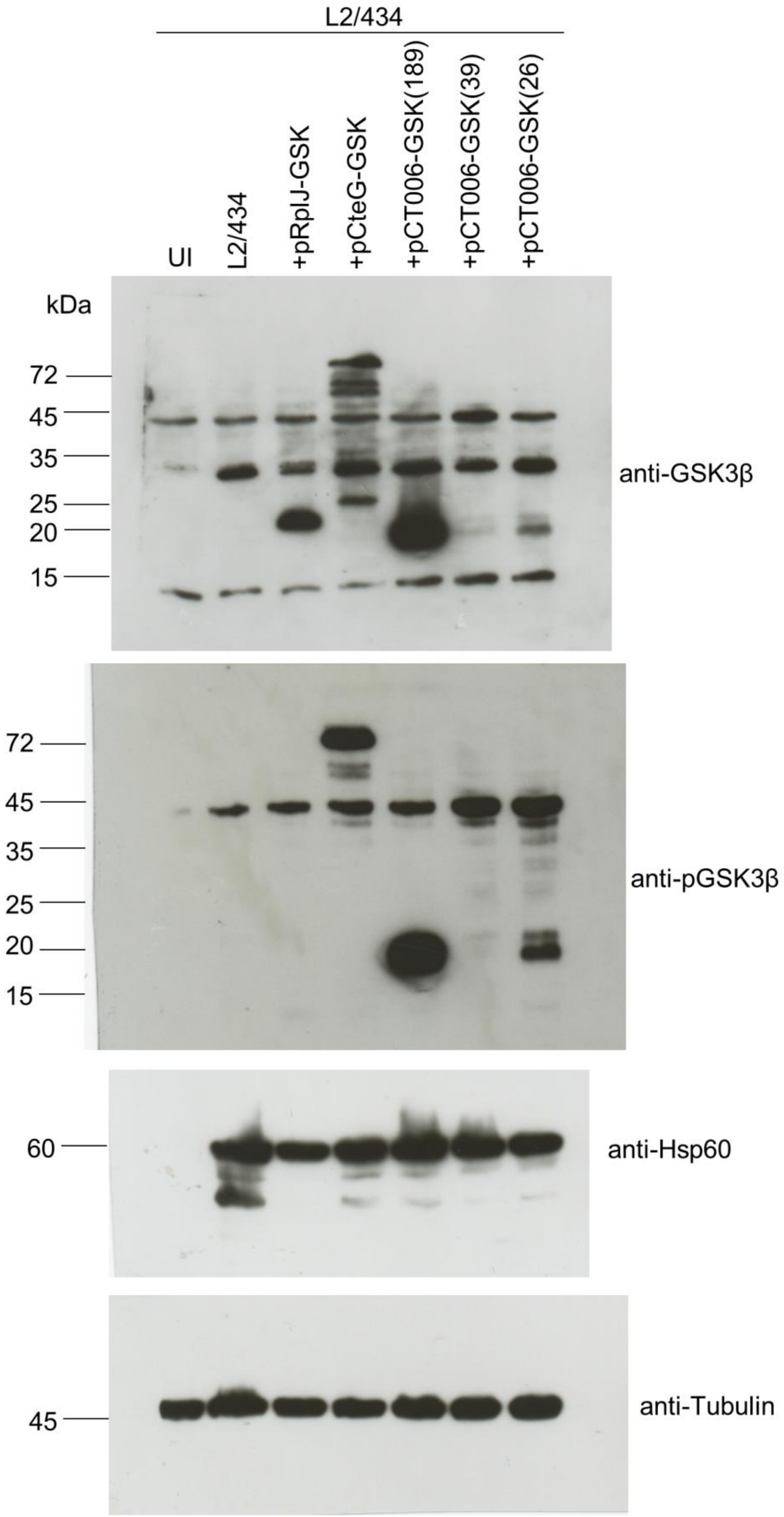

2. Uncropped blot images from Fig 6a.

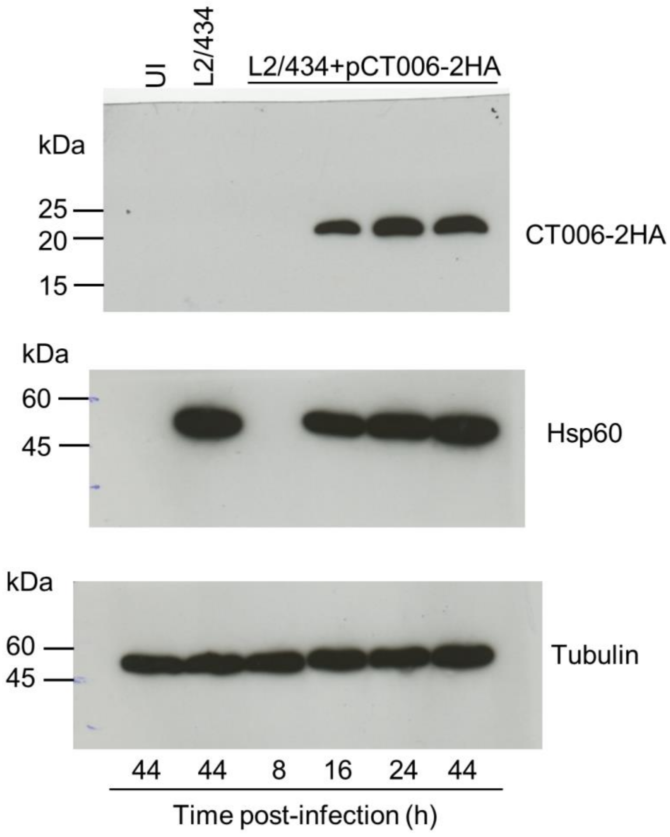

3. Uncropped blot images from Fig a in S1 Fig.

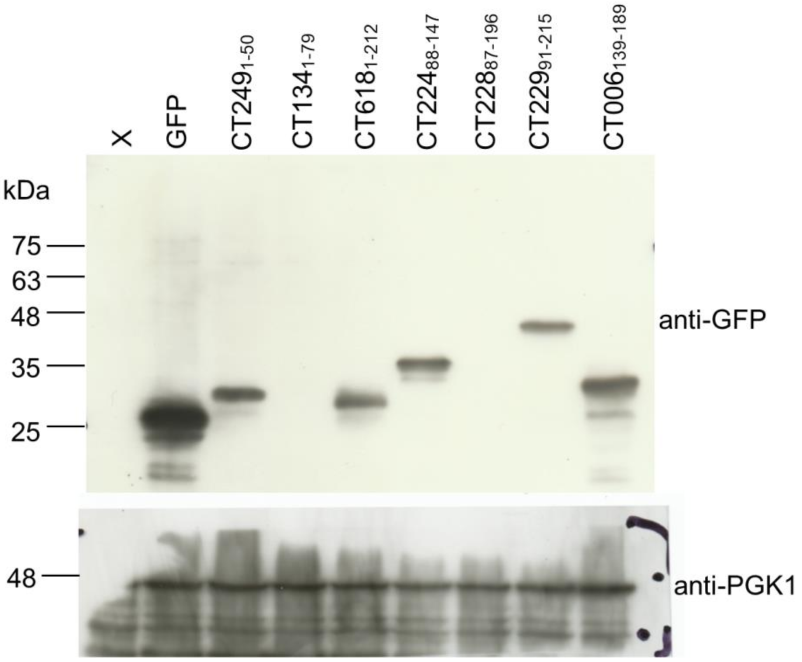

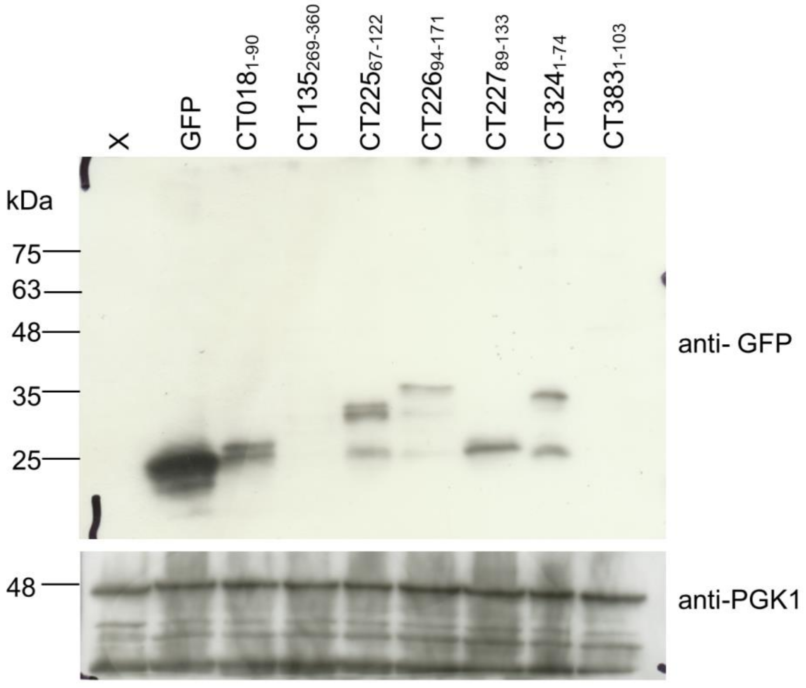

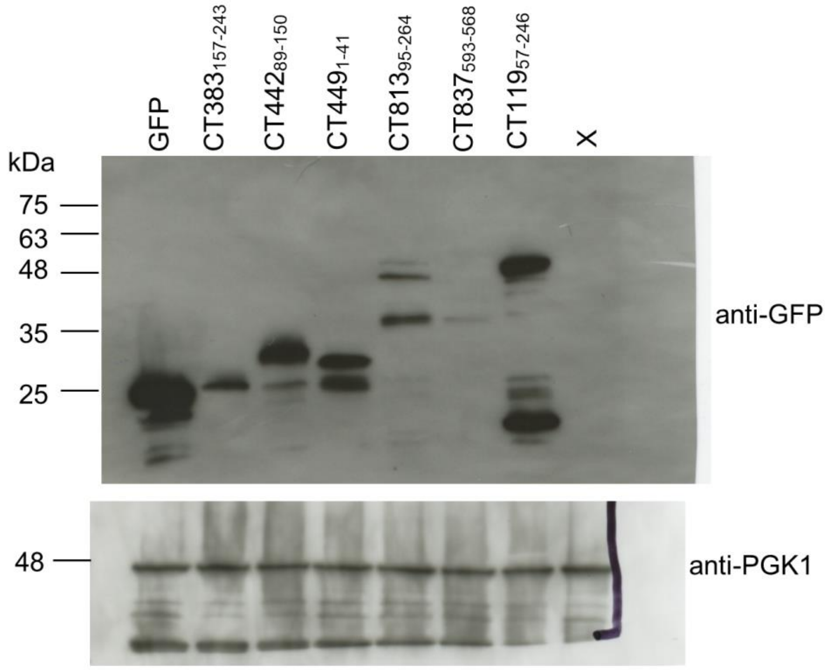
GFP
CT383157-243
CT44289-150
CT4491-41
CT81395-264
CT837593-568
CT11957-246
X
kDa
75
63
48
35
25
anti-GFP
48
anti-PGK1

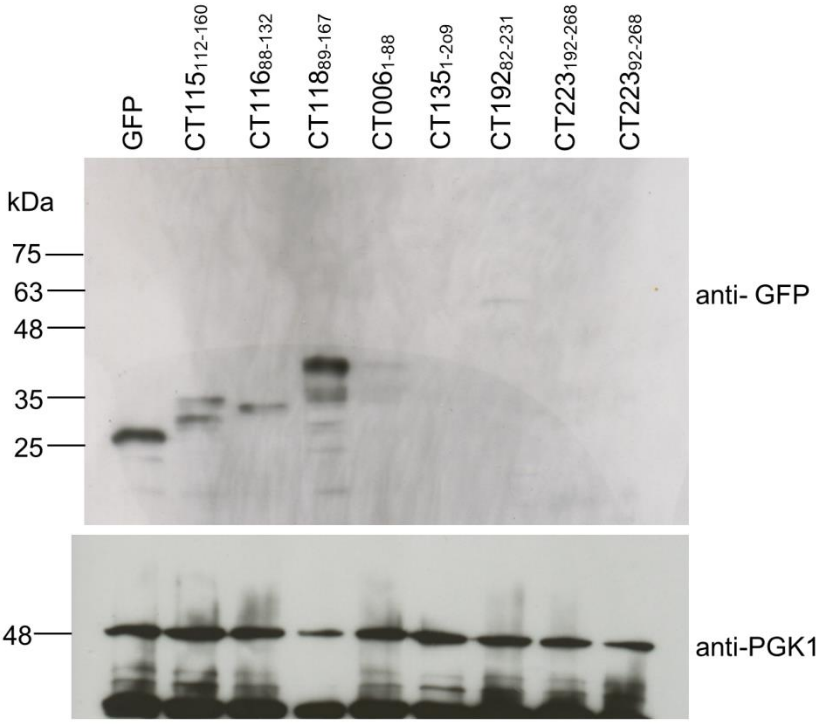
GFP
CT115112-160
CT11688-132
CT11889-167
CT0061-88
CT1351-209
CT19282-231
CT223192-268
CT22392-268
kDa
75
63
48
35
25
anti- GFP
48
anti-PGK1

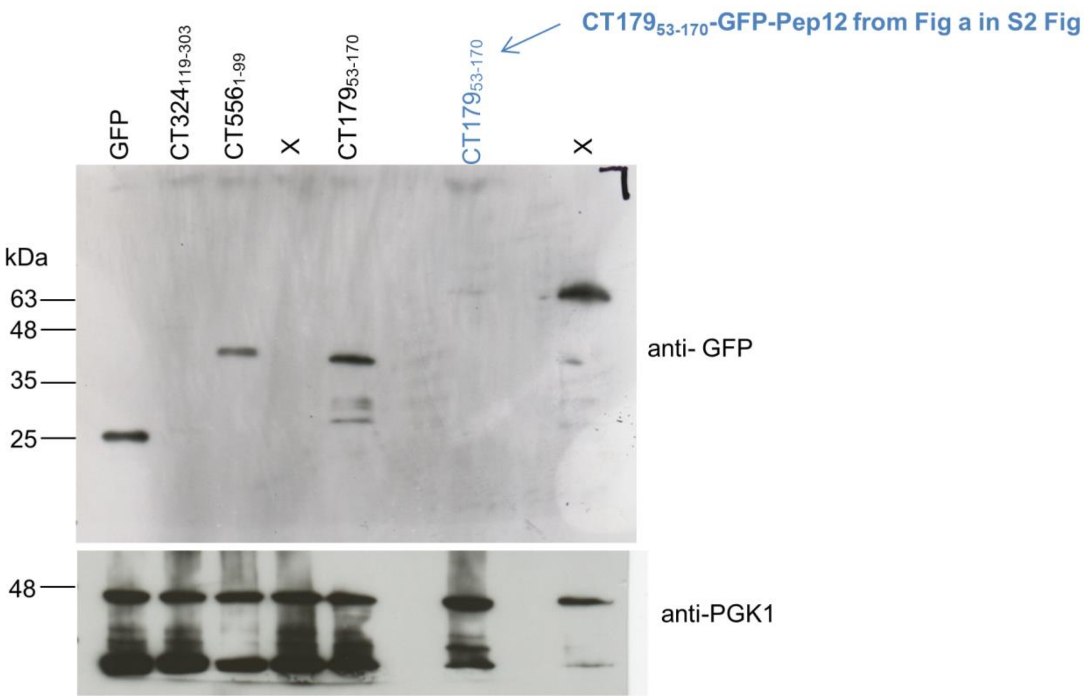
CT179$_{53-170}$-GFP-Pep12 from Fig a in S2 Fig
GFP
CT324$_{119-303}$
CT556$_{1-99}$
X
CT179$_{53-170}$
CT179$_{53-170}$
X
kDa
63
48
35
25
anti- GFP
48
anti-PGK1

4. Uncropped blot images from Fig b in S1 Fig.

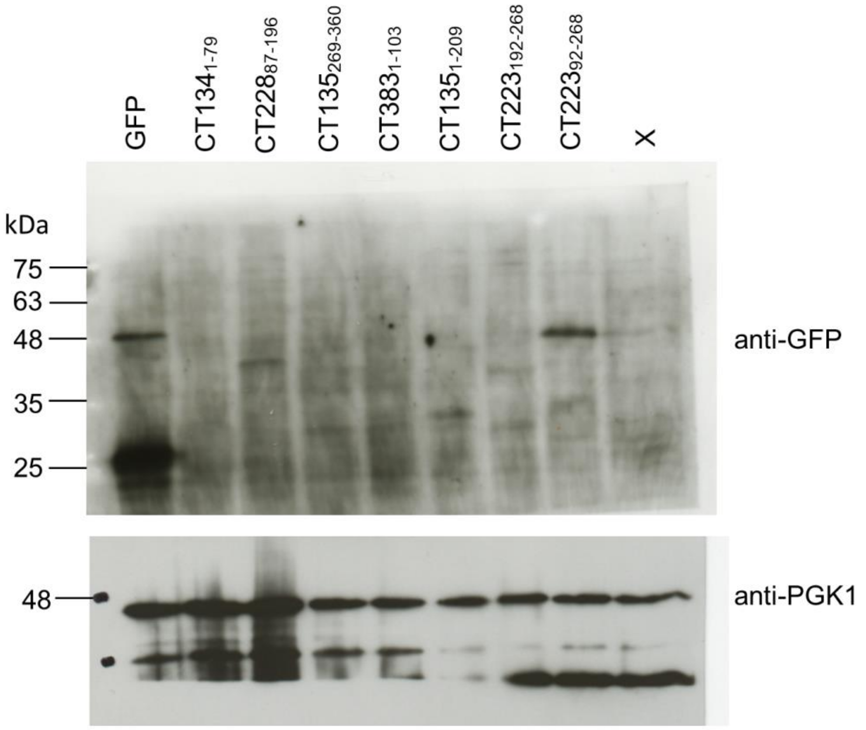

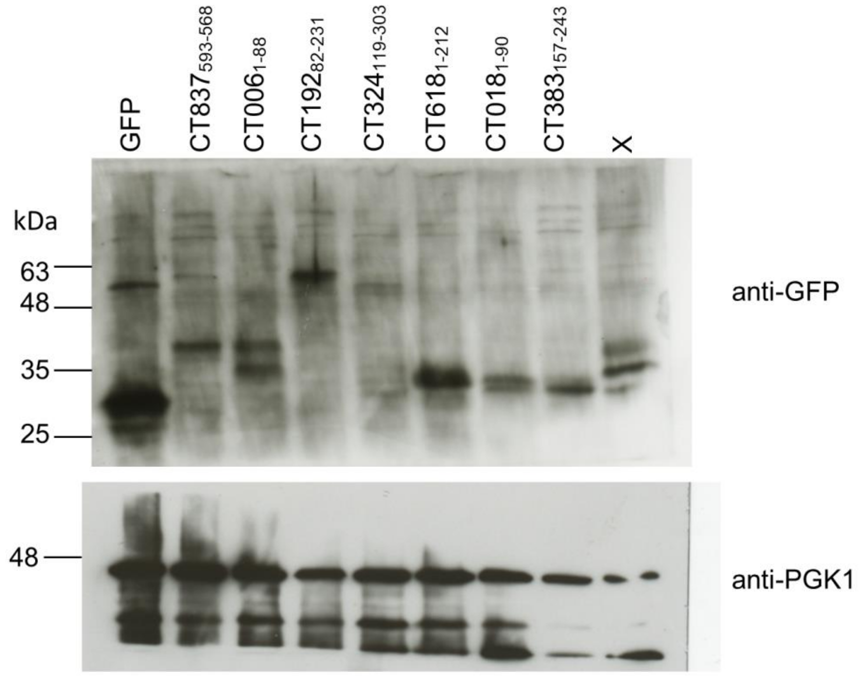

5. Uncropped blot images from Fig a in S2 Fig.

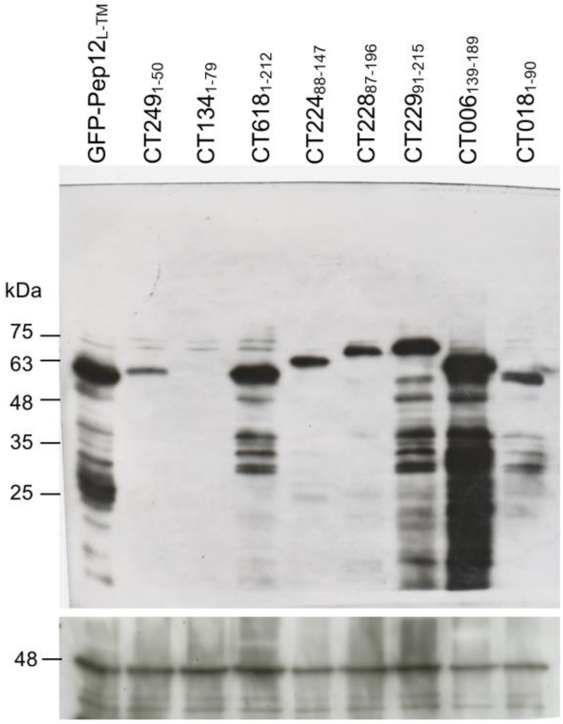

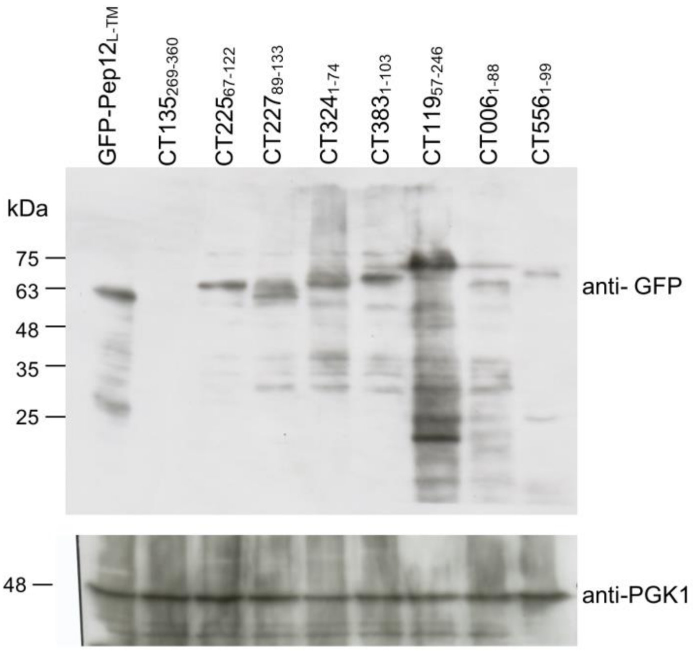

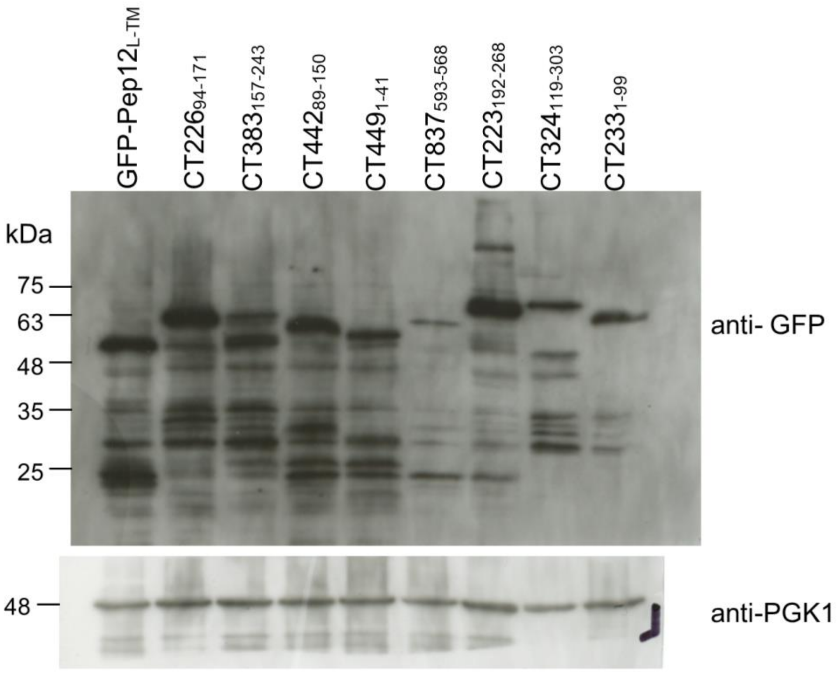

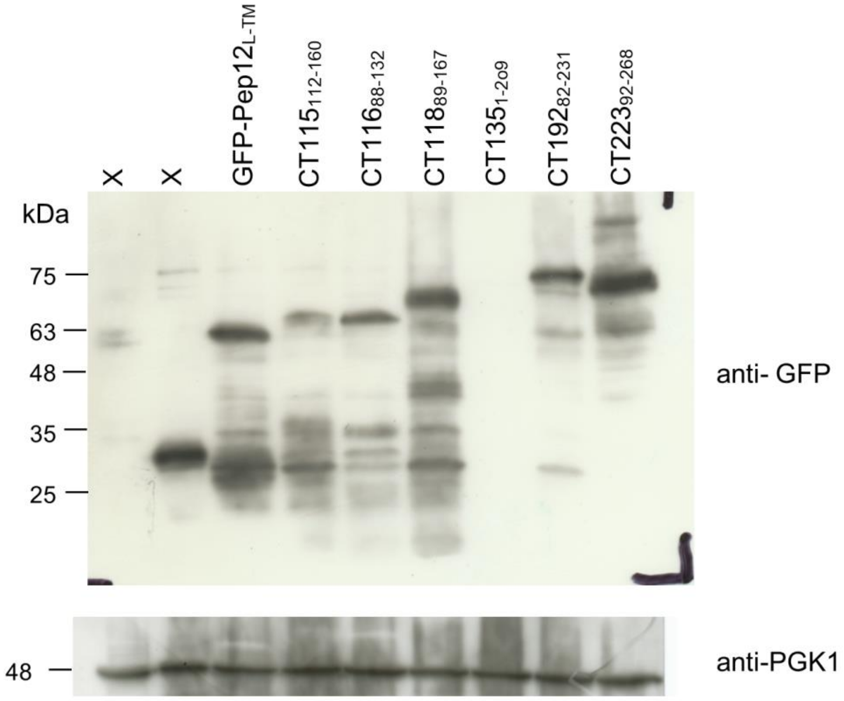

6. Uncropped blot images from Fig b in S2 Fig.

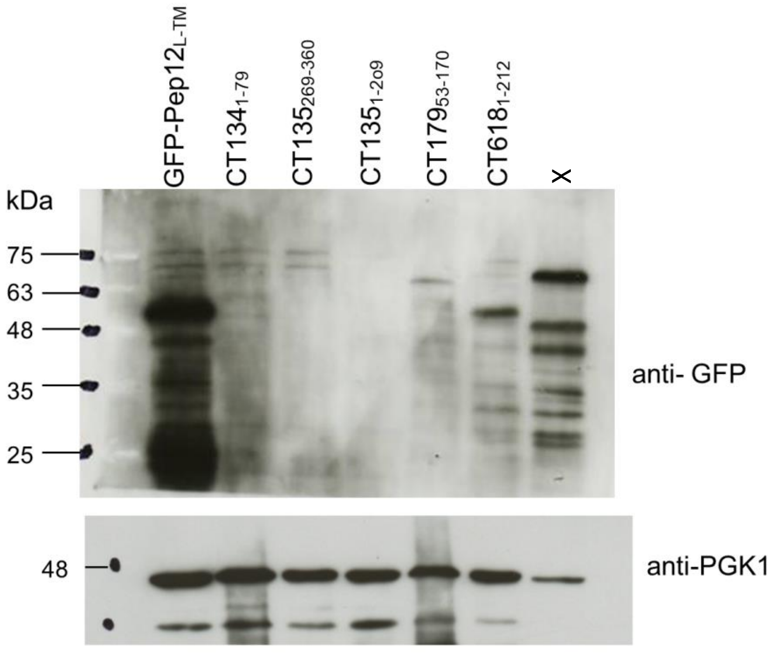

7. Uncropped blot images from Fig b in S7 Fig

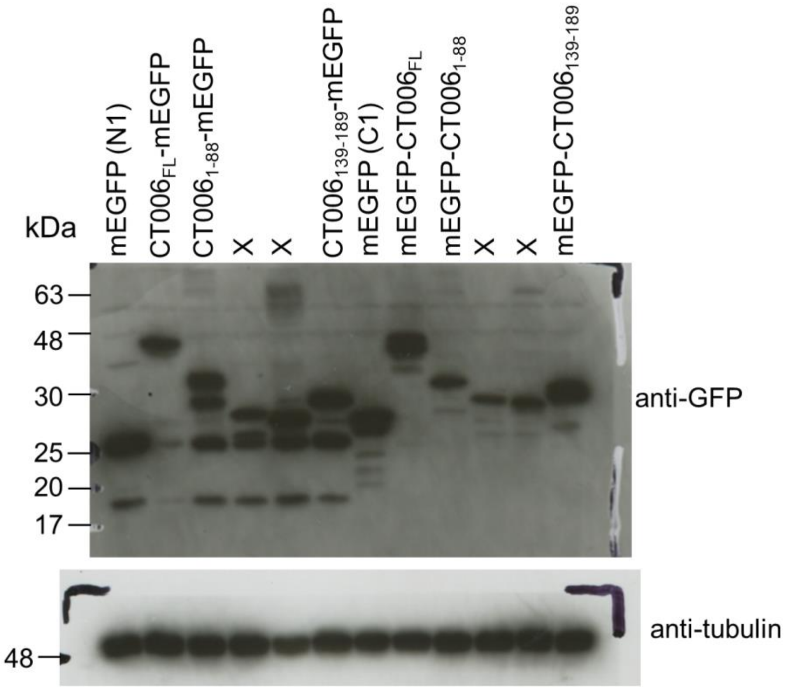

8. Uncropped blot images from S9 Fig

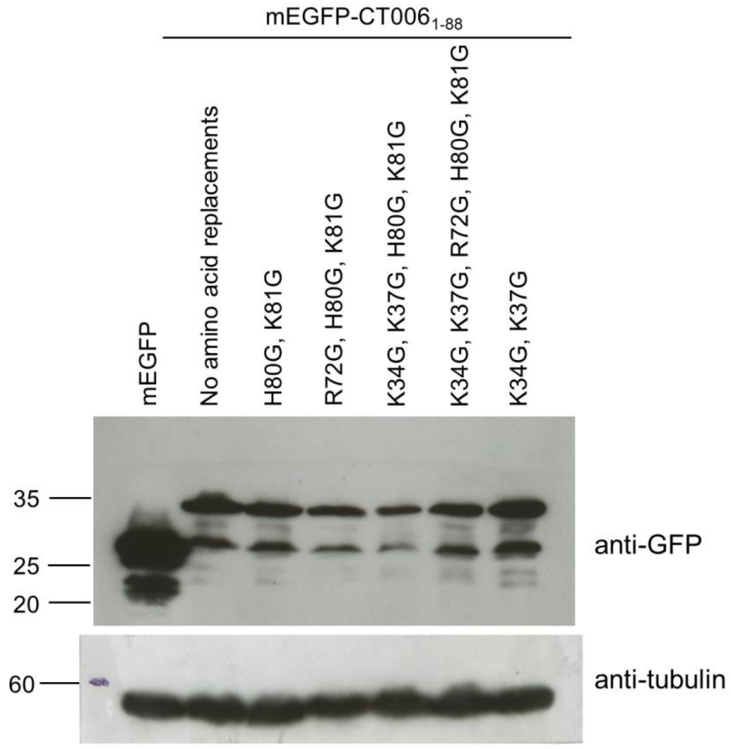

9. Uncropped blot images from Fig a in S11 Fig.

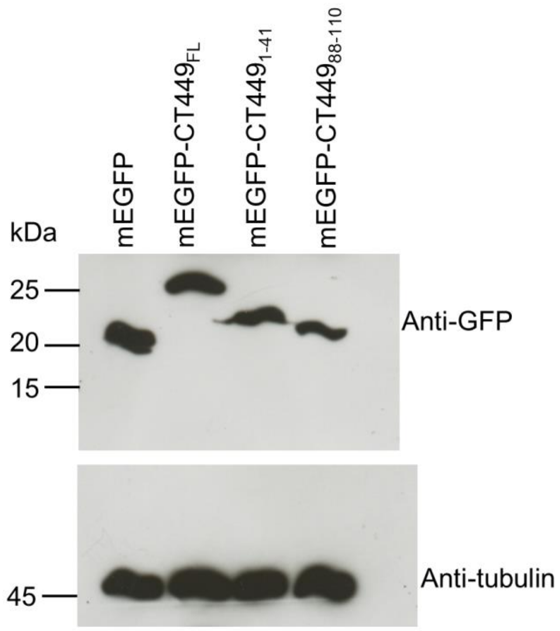

10. **Uncropped blot images from Fig a in S12 Fig.**

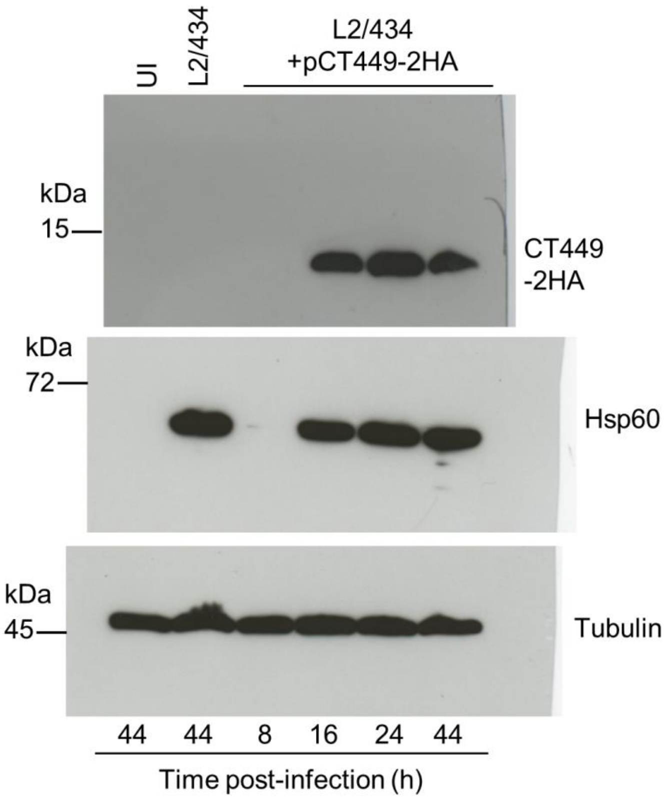

11. **Uncropped blot images from Fig b in S12 Fig.**

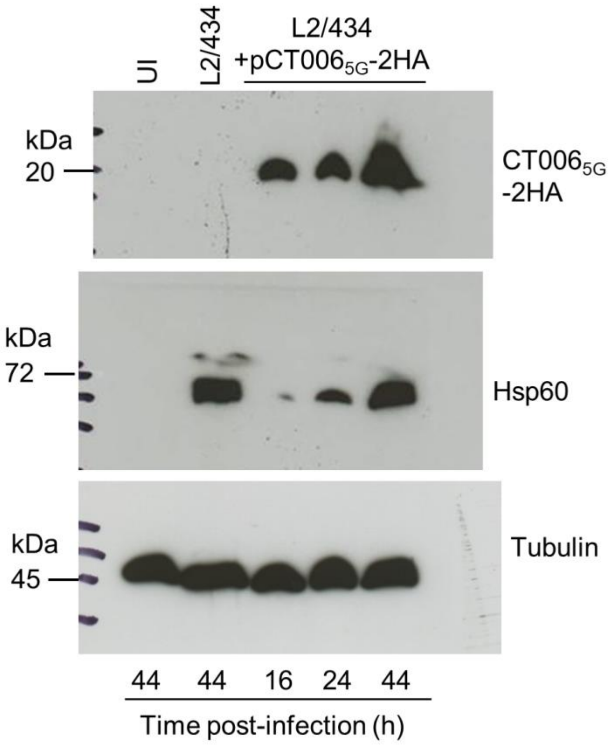

12. Uncropped blot images from Fig c in S12 Fig.

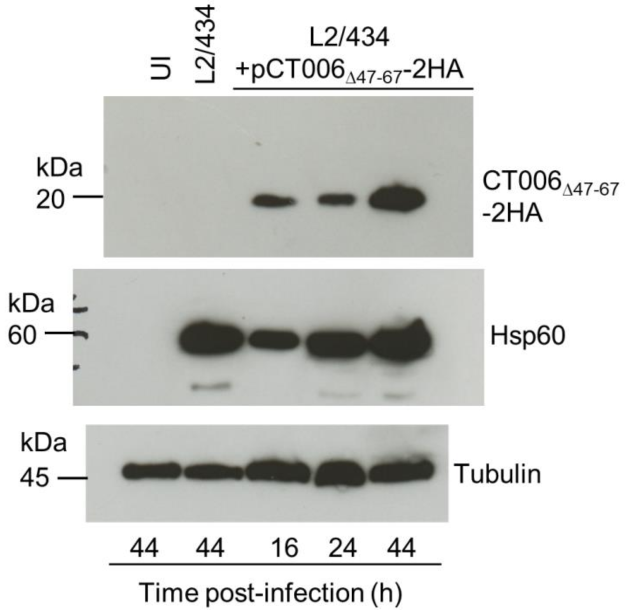

Supplement: S16 Fig — Uncroppped blot images from Figs 5a and 6a, Fig a in S1 Fig, Fig b in S1 Fig, Fig a in S2 Fig, Fig b in S2 Fig, Fig b in S7 Fig, S9 Fig, Fig a in S11 Fig, Fig a in S12 Fig, Fig b in S12 Fig, and Fig c in S12 Fig. (PDF) [file pone.0264292.s016.pdf]
