# Supplementary material for: The Chlamydia trachomatis inclusion membrane protein CT006 associates with lipid droplets in eukaryotic cells
Source: PLoS One. 2022 Feb 22;17(2):e0264292. doi: 10.1371/journal.pone.0264292 (PMC8863265; doi:10.1371/journal.pone.0264292)
Supplement: S1 Table — (PDF) [file pone.0264292.s017.pdf]

S1 Table. ***Saccharomyces cerevisiae*** **strains used in this work.**

Strains	Relevant Genotype	Source/Reference
NSY01	BHY10 diploid a/α, CPY-Inv, *inv-*, *ura-*	Shohdy *et al.*, 2005
SCIF00	NSY01 P*gal-gfp* (pKS84)	Franco *et al.*, 2012
SCIF01	NSY01 P*gal-vipA-gfp* (pIF206)	Franco *et al.*, 2012
SCNS00	NSY01 P*gal-vps4*^{E233Q}	Shohdy *et al.*, 2005
SCJNB01	NSY01 P*gal-ct249*$_{1-50}$*-gfp* (pJB27)	This work
SCJNB02	NSY01 P*gal-ct134*$_{1-79}$*-gfp* (pJB28)	This work
SCJNB03	NSY01 P*gal-ct618*$_{1-212}$*-gfp* (pJB29)	This work
SCJNB04	NSY01 P*gal-ct224*$_{88-147}$*-gfp* (pJB30)	This work
SCJNB05	NSY01 P*gal-ct228*$_{87-196}$*-gfp* (pJB31)	This work
SCJNB06	NSY01 P*gal- ct229*$_{91-215}$*-gfp* (pJB32)	This work
SCJNB07	NSY01 P*gal-ct006*$_{139-189}$*-gfp* (pJB35)	This work
SCJNB08	NSY01 P*gal-ct018*$_{1-90}$*-gfp* (pLJM1076)	This work
SCJNB09	NSY01 P*gal-ct135*$_{269-360}$*-gfp* (pJB33)	This work
SCJNB10	NSY01 P*gal-ct225*$_{67-122}$*-gfp* (pLJM1077)	This work
SCJNB11	NSY01 P*gal-ct226*$_{94-171}$*-gfp* (pJB36)	This work
SCJNB12	NSY01 P*gal-ct227*$_{89-133}$*-gfp* (pLJM1078)	This work
SCJNB13	NSY01 P*gal-ct324*$_{1-74}$*-gfp* (pJB37)	This work
SCJNB14	NSY01 P*gal-ct383*$_{1-103}$*-gfp* (pJB40)	This work
SCJNB15	NSY01 P*gal-ct383*$_{157-243}$*-gfp* (pJB34)	This work
SCJNB16	NSY01 P*gal-ct442*$_{89-150}$*-gfp* (pLJM1079)	This work
SCJNB17	NSY01 P*gal-ct449*$_{1-41}$*-gfp* (pJB38)	This work
SCJNB18	NSY01 P*gal-ct813*$_{95-264}$*-gfp* (pJB41)	This work
SCJNB19	NSY01 P*gal-ct837*$_{593-658}$*-gfp* (pJB42)	This work
SCJNB20	NSY01 P*gal-ct119*$_{57-246}$*-gfp* (pJB43)	This work
SCJNB21	NSY01 *Pgal-ct115*$_{112-160}$*-gfp* (pJB39)	This work
SCJNB22	NSY01 P*gal-ct116*$_{88-132}$*-gfp* (pJB44)	This work
SCJNB23	NSY01 P*gal-ct118*$_{89-167}$*-gfp* (pJB45)	This work
SCJNB25	NSY01 P*gal-ct006*$_{1-88}$*-gfp* (pJB46)	This work
SCJNB26	NSY01 P*gal-ct135*$_{1-209}$*-gfp* (pJB47)	This work
SCJNB27	NSY01 P*gal-ct192*$_{82-231}$*-gfp* (pJB49)	This work
SCJNB28	NSY01 P*gal-ct223*$_{192-268}$*-gfp* (pJB50)	This work
SCJNB29	NSY01 P*gal-ct223*$_{92-268}$*-gfp* (pJB48)	This work
SCJNB30	NSY01 P*gal-ct324*$_{119-303}$*-gfp* (pJB51)	This work
SCJNB31	NSY01 P*gal-ct556*$_{1-99}$*-gfp* (pJB52)	This work
SCJNB36	NSY01 P*gal-ct179*$_{53-170}$*-gfp* (pJB54)	This work
SCJNB37	NSY01 P*gal-gfp- pep12*$_{L-TM}$ (pJB55)	This work
SCJNB38	NSY01 P*gal-ct249*$_{1-50}$*-gfp-pep12*$_{L-TM}$ (pJB57)	This work
SCJNB39	NSY01 P*gal-ct134*$_{1-79}$*-gfp- pep12*$_{L-TM}$ (pJB58)	This work
SCJNB40	NSY01 P*gal-ct618*$_{1-212}$*-gfp- pep12*$_{L-TM}$ (pJB59)	This work
SCJNB41	NSY01 P*gal-ct224*$_{88-147}$*-gfp- pep12*$_{L-TM}$ (pJB60)	This work
SCJNB42	NSY01 P*gal-ct228*$_{87-196}$*-gfp- pep12*$_{L-TM}$ (pJB61)	This work
SCJNB43	NSY01 P*gal- ct229*$_{91-215}$*-gfp- pep12*$_{L-TM}$ (pJB62)	This work
SCJNB44	NSY01 P*gal-ct006*$_{139-189}$*-gfp- pep12*$_{L-TM}$ (pJB63)	This work
SCJNB45	NSY01 P*gal-ct018*$_{1-90}$*-gfp- pep12*$_{L-TM}$ (pJB64)	This work

S1 Table. ***Continued.***

Strains	Relevant Genotype	Source/Reference
SCJNB46	NSY01 P*gal-ct135*$_{269\text{-}360}$-*gfp- pep12*$_{L\text{-}TM}$ (pJB65)	This work
SCJNB47	NSY01 P*gal-ct225*$_{67\text{-}122}$-*gfp- pep12*$_{L\text{-}TM}$ (pJB66)	This work
SCJNB48	NSY01 P*gal-ct226*$_{94\text{-}171}$-*gfp- pep12*$_{L\text{-}TM}$ (pJB80)	This work
SCJNB49	NSY01 P*gal-ct227*$_{89\text{-}133}$-*gfp- pep12*$_{L\text{-}TM}$ (pJB68)	This work
SCJNB50	NSY01 P*gal-ct324*$_{1\text{-}74}$-*gfp- pep12*$_{L\text{-}TM}$ (pJB67)	This work
SCJNB51	NSY01 P*gal-ct383*$_{1\text{-}103}$-*gfp- pep12*$_{L\text{-}TM}$ (pJB69)	This work
SCJNB52	NSY01 P*gal-ct383*$_{157\text{-}243}$-*gfp- pep12*$_{L\text{-}TM}$ (pJB70)	This work
SCJNB53	NSY01 P*gal-ct442*$_{89\text{-}150}$-*gfp- pep12*$_{L\text{-}TM}$ (pJB71)	This work
SCJNB54	NSY01 P*gal-ct449*$_{1\text{-}41}$-*gfp- pep12*$_{L\text{-}TM}$ (pJB72)	This work
SCJNB56	NSY01 P*gal-ct837*$_{593\text{-}658}$-*gfp- pep12*$_{L\text{-}TM}$ (pJB74)	This work
SCJNB57	NSY01 P*gal-ct119*$_{57\text{-}246}$-*gfp- pep12*$_{L\text{-}TM}$ (pJB88)	This work
SCJNB58	NSY01 *Pgal-ct115*$_{112\text{-}160}$-*gfp- pep12*$_{L\text{-}TM}$ (pJB75)	This work
SCJNB59	NSY01 P*gal-ct116*$_{88\text{-}132}$-*gfp- pep12*$_{L\text{-}TM}$ (pJB76)	This work
SCJNB60	NSY01 P*gal-ct118*$_{89\text{-}167}$-*gfp- pep12*$_{L\text{-}TM}$ (pJB79)	This work
SCJNB61	NSY01 P*gal-ct006*$_{1\text{-}88}$-*gfp- pep12*$_{L\text{-}TM}$ (pJB81)	This work
SCJNB62	NSY01 P*gal-ct135*$_{1\text{-}209}$-*gfp- pep12*$_{L\text{-}TM}$ (pJB84)	This work
SCJNB63	NSY01 P*gal-ct192*$_{82\text{-}231}$-*gfp- pep12*$_{L\text{-}TM}$ (pJB82)	This work
SCJNB64	NSY01 P*gal-ct223*$_{192\text{-}268}$-*gfp- pep12*$_{L\text{-}TM}$ (pJB77)	This work
SCJNB65	NSY01 P*gal-ct223*$_{92\text{-}268}$-*gfp- pep12*$_{L\text{-}TM}$ (pJB78)	This work
SCJNB66	NSY01 P*gal-ct324*$_{119\text{-}303}$-*gfp- pep12*$_{L\text{-}TM}$ (pJB83)	This work
SCJNB67	NSY01 P*gal-ct556*$_{1\text{-}99}$-*gfp- pep12*$_{L\text{-}TM}$ (pJB85)	This work
SCJNB70	NSY01 P*gal-ct233*$_{1\text{-}99}$-*gfp- pep12*$_{L\text{-}TM}$ (pJB86)	This work
SCJNB72	NSY01 P*gal-ct179*$_{53\text{-}170}$-*gfp- pep12*$_{L\text{-}TM}$ (pJB87)	This work
YPH499	*MATa ura3-52 lys2-801_amber ade2-101_ochre trp1-Δ63 his3-Δ200 leu2-Δ1*	Kindly provided by Victor J. Cid
SCJNB79	YPH499 P*gal-gfp* (pKS84) + (pGreg505 Erg6-mCherry)	This work
SCJNB80	YPH499 P*gal-ct006*$_{1\text{-}88}$-*gfp* (pJB46) + (pGreg505 Erg6-mCherry)	This work
SCJNB82	YPH499 P*gal-ct006*$_{139\text{-}189}$-*gfp* (pJB35) + (pGreg505 Erg6-mCherry)	This work

S1 Table References

Franco IS, Shohdy N, Shuman HA. The Legionella pneumophila effector VipA is an actin nucleator that alters host cell organelle trafficking. PLoS Pathog. 2012;8(2):e1002546.

Shohdy N, Efe JA, Emr SD, Shuman HA. Pathogen effector protein screening in yeast identifies Legionella factors that interfere with membrane trafficking. Proc Natl Acad Sci U S A. 2005;102(13):4866–4871.
