# Supplementary material for: The Chlamydia trachomatis inclusion membrane protein CT006 associates with lipid droplets in eukaryotic cells
Source: PLoS One. 2022 Feb 22;17(2):e0264292. doi: 10.1371/journal.pone.0264292 (PMC8863265; doi:10.1371/journal.pone.0264292)
Supplement: S2 Table — (PDF) [file pone.0264292.s018.pdf]

**S2 Table. Inc-GFP fusion proteins - summary of production and localization in *Saccharomyces cerevisiae*, and induction of a vacuolar protein sorting (Vps) defect.**

| Inc (aa-aa) | Produced | Subcellular localization | Vps defect |
|---|---|---|---|
| CT249 (1-50) | Yes | Cytosolic | No |
| CT134 (1-79) | No | Not detected | No |
| CT618 (1-212) | Below Mw | Mitochondria-like puncta | No |
| CT224 (88-147) | Yes | Cytosolic | No |
| CT228 (87-196) | No | Not detected | No |
| CT229 (91-215) | Yes | Endosomes | Yes |
| CT006 (139-189) | Yes | Cytosolic | No |
| CT018 (1-90) | Below Mw | Mitochondria-like puncta | No |
| CT135 (269-360) | No | Not detected | No |
| CT225 (67-122) | Yes | Not detected | No |
| CT226 (94-171) | Yes | Not detected | No |
| CT227 (89-133) | Yes | Cytosolic | No |
| CT324 (1-74) | Yes | Not detected | No |
| CT383 (1-103) | No | Not detected | No |
| CT383 (157-243) | Below Mw | Mitochondria-like puncta | No |
| CT442 (89-150) | Yes | Cytosolic | No |
| CT449 (1-41) | Yes | Cytosolic | No |
| CT813 (95-264) | Yes | Not detected | No |
| CT837 (593-568) | Yes | Not detected | No |
| CT119 (57-246) | Yes | Cytosolic | No |
| CT115 (112-160) | Yes | Cytosolic | No |
| CT116 (88-132) | Yes | Cytosolic | No |
| CT118 (89-167) | Yes | Cytosolic | No |
| CT006 (1-88) | Yes | Lipid droplets | No |
| CT135 (1-209) | No | Not detected | No |
| CT192 (82-231) | Yes | Cytosolic | No |
| CT223 (192-268) | Yes | Not detected | No |
| CT223 (92-268) | Yes | Not detected | No |
| CT324 (119-303) | Yes | Mitochondria-like puncta | No |
| CT556 (1-99) | Yes | Cytosolic | No |
| CT233 (1-99) | Not tested | Not tested | Not tested |
| CT179 (53-170) | Yes | Mitochondria-like puncta | No |
