# Supplementary material for: The Chlamydia trachomatis inclusion membrane protein CT006 associates with lipid droplets in eukaryotic cells
Source: PLoS One. 2022 Feb 22;17(2):e0264292. doi: 10.1371/journal.pone.0264292 (PMC8863265; doi:10.1371/journal.pone.0264292)
Supplement: S3 Table — (PDF) [file pone.0264292.s019.pdf]

S3 Table. Inc-GFP-Pep12$_{L-TM}$ fusion proteins - summary of production and localization in *Saccharomyces cerevisiae*, and induction of a vacuolar protein sorting (Vps) defect.

Inc (aa-aa)	Produced	Subcellular localization	Vps defect
CT249 (1-50)	Yes	Puncta	No
CT134 (1-79)	No	Puncta	No
CT618 (1-212)	Below Mw	Puncta	No
CT224 (88-147)	Yes	Vacuoles+puncta	No
CT228 (87-196)	Yes	Puncta	No
CT229 (91-215)	Yes	Puncta	No
CT006 (139-189)	Yes	Vacuoles+puncta	No
CT018 (1-90)	Yes	Puncta	No
CT135 (269-360)	No	Not detected	No
CT225 (67-122)	Yes	Vacuoles+puncta	No
CT226 (94-171)	Yes	Vacuoles+puncta	No
CT227 (89-133)	Yes	Puncta	No
CT324 (1-74)	Yes	Vacuoles+puncta	No
CT383 (1-103)	Yes	Puncta	No
CT383 (157-243)	Yes	Puncta	No
CT442 (89-150)	Yes	Vacuoles+puncta	No
CT449 (1-41)	Yes	Vacuoles+puncta	No
CT813 (95-264)	Not tested	Not tested	No
CT837 (593-568)	Yes	Not detected	No
CT119 (57-246)	Yes	Not detected	No
CT115 (112-160)	Yes	Vacuoles	No
CT116 (88-132)	Yes	Vacuoles	No
CT118 (89-167)	Yes	Vacuoles	No
CT006 (1-88)	Yes	Vacuoles+puncta	No
CT135 (1-209)	No	Not detected	Not tested
CT192 (82-231)	Yes	Vacuoles+puncta	No
CT223 (192-268)	Yes	Puncta	Yes
CT223 (92-268)	Yes	Puncta	Yes
CT324 (119-303)	Below Mw	Puncta	No
CT556 (1-99)	Yes	Puncta	No
CT179 (53-170)	Yes	Puncta	No
