# Supplementary material for: The Chlamydia trachomatis inclusion membrane protein CT006 associates with lipid droplets in eukaryotic cells
Source: PLoS One. 2022 Feb 22;17(2):e0264292. doi: 10.1371/journal.pone.0264292 (PMC8863265; doi:10.1371/journal.pone.0264292)
Supplement: S4 Table — (PDF) [file pone.0264292.s020.pdf]

S4 Table. Plasmids used in this work.

Plasmid	Description	Source/Reference
	**Plasmids for production of proteins in *Saccharomyces cerevisiae***	
pGreg505	Erg6-mCherry; (Amp^R)	Kindly provided by Roger Schneiter. Khaddaj *et al.*, 2022
pKS84	GFP; pKS84 derivatives were used for ectopic production of GFP fusion proteins in *Saccharomyces cerevisiae* from a galactose-inducible promoter (Amp^R)	De Felipe *et al.*, 2008
pIF206	VipA-GFP	Franco *et al.*, 2012
pJB27	$CT249_{1-50}$-GFP; $ct249_{1-50}$ was amplified from *C. trachomatis* L2/434 chromosomal DNA by PCR with oligos 1977 and 1978, digested with BamHI-HindIII and inserted in the same sites of pKS84.	This work
pJB28	$CT134_{1-79}$-GFP; $ct134_{1-79}$ was amplified from *C. trachomatis* L2/434 chromosomal DNA by PCR with oligos 1979 and 1980, digested with BamHI-HindIII and inserted in the same sites of pKS84.	This work
pJB29	$CT618_{1-212}$-GFP; $ct618_{1-212}$ was amplified from *C. trachomatis* L2/434 chromosomal DNA by PCR with oligos 1981 and 1982, digested with BamHI-HindIII and inserted in the same sites of pKS84.	This work
pJB30	$CT224_{88-147}$-GFP; $ct224_{88-147}$ was amplified from *C. trachomatis* L2/434 chromosomal DNA by PCR with oligos 1983 and 1984 and inserted by restriction-free cloning in pKS84.	This work
pJB31	$CT228_{87-196}$-GFP; $ct228_{87-196}$ was amplified from *C. trachomatis* L2/434 chromosomal DNA by PCR with oligos 1985 and 1986 and inserted by restriction-free cloning in pKS84.	This work
pJB32	$CT229_{91-215}$-GFP; $ct229_{91-215}$ was amplified from *C. trachomatis* L2/434 chromosomal DNA by PCR with oligos 1987 and 1988 and inserted by restriction-free cloning in pKS84.	This work
pJB33	$CT135_{269-360}$-GFP; $ct135_{269-360}$ was amplified from *C. trachomatis* L2/434 chromosomal DNA by PCR with oligos 2002 and 2003, digested with BamHI-HindIII and inserted in the same sites of pKS84.	This work
pJB34	$CT383_{157-243}$-GFP; $ct383_{157-243}$ was amplified from *C. trachomatis* L2/434 chromosomal DNA by PCR with oligos 2014 and 2015, digested with BamHI-HindIII and inserted in the same sites of pKS84.	This work
pJB35	$CT006_{139-189}$-GFP; $ct006_{139-189}$ was amplified from *C. trachomatis* L2/434 chromosomal DNA by PCR with oligos 1998 and 1999, digested with BamHI-HindIII and inserted in the same sites of pKS84.	This work
pJB36	$CT226_{94-171}$-GFP; $ct226_{94-171}$ was amplified from *C. trachomatis* L2/434 chromosomal DNA by PCR with oligos 2006 and 2007, digested with BamHI-HindIII and inserted in the same sites of pKS84.	This work

S4 Table. ***Continued.***

Plasmid	Description	Source/Reference
pJB37	CT324$_{1\text{-}74}$-GFP; *ct324$_{1\text{-}74}$* was amplified from *C. trachomatis* L2/434 chromosomal DNA by PCR with oligos 2010 and 2011, digested with BamHI-HindIII and inserted in the same sites of pKS84.	This work
pJB38	CT449$_{1\text{-}41}$-GFP; *ct449$_{1\text{-}41}$* was amplified from *C. trachomatis* L2/434 chromosomal DNA by PCR with oligos 2018 and 2019, digested with BamHI-HindIII and inserted in the same sites of pKS84.	This work
pJB39	CT115$_{112\text{-}160}$-GFP; *ct115$_{112\text{-}160}$* was amplified from *C. trachomatis* L2/434 chromosomal DNA by PCR with oligos 2026 and 2027, digested with BamHI-HindIII and inserted in the same sites of pKS84.	This work
pJB40	CT383$_{1\text{-}103}$-GFP; *ct383$_{1\text{-}103}$* was amplified from *C. trachomatis* L2/434 chromosomal DNA by PCR with oligos 2012 and 2013, digested with BamHI-HindIII and inserted in the same sites of pKS84.	This work
pJB41	CT813$_{95\text{-}264}$-GFP; *ct813$_{95\text{-}264}$* was amplified from *C. trachomatis* L2/434 chromosomal DNA by PCR with oligos 2020 and 2021, digested with BamHI-HindIII and inserted in the same sites of pKS84.	This work
pJB42	CT837$_{593\text{-}658}$-GFP; *ct837$_{593\text{-}658}$* was amplified from *C. trachomatis* L2/434 chromosomal DNA by PCR with oligos 2022 and 2023, digested with BamHI-HindIII and inserted in the same sites of pKS84.	This work
pJB43	CT119$_{57\text{-}246}$-GFP; *ct119$_{57\text{-}246}$* was amplified from *C. trachomatis* L2/434 chromosomal DNA by PCR with oligos 2024 and 2025, digested with BamHI-HindIII and inserted in the same sites of pKS84.	This work
pJB44	CT116$_{88\text{-}132}$-GFP; *ct116$_{88\text{-}132}$* was amplified from *C. trachomatis* L2/434 chromosomal DNA by PCR with oligos 2028 and 2029, digested with BamHI-HindIII and inserted in the same sites of pKS84.	This work
pJB45	CT118$_{89\text{-}167}$-GFP; *ct118$_{89\text{-}167}$* was amplified from *C. trachomatis* L2/434 chromosomal DNA by PCR with oligos 2030 and 2031, digested with BamHI-HindIII and inserted in the same sites of pKS84.	This work
pJB46	CT006$_{1\text{-}88}$-GFP; *ct006$_{1\text{-}88}$* was amplified from *C. trachomatis* L2/434 chromosomal DNA by PCR with oligos 2048 and 2049 and inserted by restriction-free cloning in pKS84.	This work
pJB47	CT135$_{1\text{-}209}$-GFP; *ct135$_{1\text{-}209}$* was amplified from *C. trachomatis* L2/434 chromosomal DNA by PCR with oligos 2050 and 2051 and inserted by restriction-free cloning in pKS84.	This work
pJB48	CT223$_{92\text{-}268}$-GFP; *ct223$_{92\text{-}268}$* was amplified from *C. trachomatis* L2/434 chromosomal DNA by PCR with oligos 2055 and 2056 and inserted by restriction-free cloning in pKS84.	This work
pJB49	CT192$_{82\text{-}231}$-GFP; *ct192$_{82\text{-}231}$* was amplified from *C. trachomatis* L2/434 chromosomal DNA by PCR with oligos 2052 and 2053 and inserted by restriction-free cloning in pKS84.	This work

S4 Table. *Continued.*

Plasmid	Description	Source/Reference
pJB50	CT223$_{192\text{-}268}$-GFP; *ct223$_{192\text{-}268}$* was amplified from *C. trachomatis* L2/434 chromosomal DNA by PCR with oligos 2054 and 2055 and inserted by restriction-free cloning in pKS84.	This work
pJB51	CT324$_{119\text{-}303}$-GFP; *ct324$_{119\text{-}303}$* was amplified from *C. trachomatis* L2/434 chromosomal DNA by PCR with oligos 2057 and 2058 and inserted by restriction-free cloning in pKS84.	This work
pJB52	CT556$_{1\text{-}99}$-GFP; *ct556$_{1\text{-}99}$* was amplified from *C. trachomatis* L2/434 chromosomal DNA by PCR with oligos 2059 and 2060 and inserted by restriction-free cloning in pKS84.	This work
pJB54	CT179$_{53\text{-}170}$-GFP; *ct179$_{53\text{-}170}$* was amplified from *C. trachomatis* L2/434 chromosomal DNA by PCR with oligos 2069 and 2070 and inserted by restriction-free cloning in pKS84.	This work
pLJM1076	CT018$_{1\text{-}90}$-GFP; *ct018$_{1\text{-}90}$* was amplified from *C. trachomatis* L2/434 chromosomal DNA by PCR with oligos 2000 and 2001, digested with BamHI-HindIII and inserted in the same sites of pKS84.	This work
pLJM1077	CT225$_{67\text{-}122}$-GFP; *ct225$_{67\text{-}122}$* was amplified from *C. trachomatis* L2/434 chromosomal DNA by PCR with oligos 2004 and 2005, digested with BamHI-HindIII and inserted in the same sites of pKS84.	This work
pLJM1078	CT227$_{89\text{-}133}$-GFP; *ct227$_{89\text{-}133}$* was amplified from *C. trachomatis* L2/434 chromosomal DNA by PCR with oligos 2008 and 2009, digested with BamHI-HindIII and inserted in the same sites of pKS84.	This work
pLJM1079	CT442$_{89\text{-}150}$-GFP; *ct442$_{89\text{-}150}$* was amplified from *C. trachomatis* L2/434 chromosomal DNA by PCR with oligos 2016 and 2017, digested with BamHI-HindIII and inserted in the same sites of pKS84.	This work
pSDY-1	GFP-Pep12$_{L\text{-}TM}$; plasmid used to amplify Pep12$_{L\text{-}TM}$.	Kindly provided by Raphael Valdivia. Sisko *et al.*, 2006
pJB55	GFP-Pep12$_{L\text{-}TM}$; *pep12$_{L\text{-}TM}$* was amplified from pSDY-1 with oligos 2046 and 2047 and inserted by restriction-free cloning in pKS84.	This work
pJB57	CT249$_{1\text{-}50}$-GFP-Pep12$_{L\text{-}TM}$; *pep12$_{L\text{-}TM}$* was amplified from pSDY-1 by PCR with oligos 2046 and 2047 and inserted by restriction-free cloning in pJB27.	This work
pJB58	CT134$_{1\text{-}79}$-GFP-Pep12$_{L\text{-}TM}$; *pep12$_{L\text{-}TM}$* was amplified from pSDY-1 by PCR with oligos 2046 and 2047 and inserted by restriction-free cloning in pJB28.	This work
pJB59	CT618$_{1\text{-}212}$-GFP-Pep12$_{L\text{-}TM}$; *pep12$_{L\text{-}TM}$* was amplified from pSDY-1 by PCR with oligos 2046 and 2047 and inserted by restriction-free cloning in pJB29.	This work
pJB60	CT224$_{88\text{-}147}$-GFP-Pep12$_{L\text{-}TM}$; *pep12$_{L\text{-}TM}$* was amplified from pSDY-1 by PCR with oligos 2046 and 2047 and inserted by restriction-free cloning in pJB30.	This work

S4 Table. ***Continued.***

Plasmid	Description	Source/Reference
pJB61	CT228$_{87\text{-}196}$-GFP-Pep12$_{L\text{-}TM}$; *pep12*$_{L\text{-}TM}$ was amplified from pSDY-1 by PCR with oligos 2046 and 2047 and inserted by restriction-free cloning in pJB31.	This work
pJB62	CT229$_{91\text{-}215}$-GFP-Pep12$_{L\text{-}TM}$; *pep12*$_{L\text{-}TM}$ was amplified from pSDY-1 by PCR with oligos 2046 and 2047 and inserted by restriction-free cloning in pJB32.	This work
pJB63	CT006$_{139\text{-}189}$-GFP-Pep12$_{L\text{-}TM}$; *pep12*$_{L\text{-}TM}$ was amplified from pSDY-1 by PCR with oligos 2046 and 2047 and inserted by restriction-free cloning in pJB35.	This work
pJB64	CT018$_{1\text{-}90}$-GFP-Pep12$_{L\text{-}TM}$; *pep12*$_{L\text{-}TM}$ was amplified from pSDY-1 by PCR with oligos 2046 and 2047 and inserted by restriction-free cloning in pLJM1076.	This work
pJB65	CT135$_{269\text{-}360}$-GFP-Pep12$_{L\text{-}TM}$; *pep12*$_{L\text{-}TM}$ was amplified from pSDY-1 by PCR with oligos 2046 and 2047 and inserted by restriction-free cloning in pJB33.	This work
pJB66	CT225$_{67\text{-}122}$-GFP-Pep12$_{L\text{-}TM}$; *pep12*$_{L\text{-}TM}$ was amplified from pSDY-1 by PCR with oligos 2046 and 2047 and inserted by restriction-free cloning in pLJM1077.	This work
pJB67	CT324$_{1\text{-}74}$-GFP-Pep12$_{L\text{-}TM}$; *pep12*$_{L\text{-}TM}$ was amplified from pSDY-1 by PCR with oligos 2046 and 2047 and inserted by restriction-free cloning in pJB37.	This work
pJB68	CT227$_{89\text{-}133}$-GFP-Pep12$_{L\text{-}TM}$; *pep12*$_{L\text{-}TM}$ was amplified from pSDY-1 by PCR with oligos 2046 and 2047 and inserted by restriction-free cloning in pLJM1078.	This work
pJB69	CT383$_{1\text{-}103}$-GFP-Pep12$_{L\text{-}TM}$; *pep12*$_{L\text{-}TM}$ was amplified from pSDY-1 by PCR with oligos 2046 and 2047 and inserted by restriction-free cloning in pJB40.	This work
pJB70	CT383$_{157\text{-}243}$-GFP-Pep12$_{L\text{-}TM}$; *pep12*$_{L\text{-}TM}$ was amplified from pSDY-1 by PCR with oligos 2046 and 2047 and inserted by restriction-free cloning in pJB34.	This work
pJB71	CT442$_{89\text{-}150}$-GFP-Pep12$_{L\text{-}TM}$; *ct442*$_{89\text{-}150}$ was amplified from pLJM1079 by PCR with oligos 2162 and 2163 and inserted by restriction-free cloning in pJB55.	This work
pJB72	CT449$_{1\text{-}41}$-GFP-Pep12$_{L\text{-}TM}$; *pep12*$_{L\text{-}TM}$ was amplified from pSDY-1 by PCR with oligos 2046 and 2047 and inserted by restriction-free cloning in pJB38.	This work
pJB74	CT837$_{593\text{-}658}$-GFP-Pep12$_{L\text{-}TM}$; *pep12*$_{L\text{-}TM}$ was amplified from pSDY-1 by PCR with oligos 2046 and 2047 and inserted by restriction-free cloning in pJB42.	This work
pJB75	CT115$_{112\text{-}160}$-GFP-Pep12$_{L\text{-}TM}$; *pep12*$_{L\text{-}TM}$ was amplified from pSDY-1 by PCR with oligos 2046 and 2047 and inserted by restriction-free cloning in pJB39.	This work
pJB76	CT116$_{88\text{-}132}$-GFP-Pep12$_{L\text{-}TM}$; *pep12*$_{L\text{-}TM}$ was amplified from pSDY-1 by PCR with oligos 2046 and 2047 and inserted by restriction-free cloning in pJB44.	This work

S4 Table. ***Continued.***

Plasmid	Description	Source/Reference
pJB77	$CT223_{192\text{-}268}$-GFP-$Pep12_{L\text{-}TM}$; *pep12*$_{L\text{-}TM}$ was amplified from pSDY-1 by PCR with oligos 2046 and 2047 and inserted by restriction-free cloning in pJB50.	This work
pJB78	$CT223_{92\text{-}268}$-GFP-$Pep12_{L\text{-}TM}$; *pep12*$_{L\text{-}TM}$ was amplified from pSDY-1 by PCR with oligos 2046 and 2047 and inserted by restriction-free cloning in pJB48.	This work
pJB79	$CT118_{89\text{-}167}$-GFP-$Pep12_{L\text{-}TM}$; *pep12*$_{L\text{-}TM}$ was amplified from pSDY-1 by PCR with oligos 2046 and 2047 and inserted by restriction-free cloning in pJB45.	This work
pJB80	$CT226_{94\text{-}171}$-GFP-$Pep12_{L\text{-}TM}$; *pep12*$_{L\text{-}TM}$ was amplified from pSDY-1 by PCR with oligos 2046 and 2047 and inserted by restriction-free cloning in pJB36.	This work
pJB81	$CT006_{1\text{-}88}$-GFP-$Pep12_{L\text{-}TM}$; *ct006*$_{1\text{-}88}$ was amplified from pJB46 by PCR with oligos 2048 and 2049 and inserted by restriction-free cloning in pJB55.	This work
pJB82	$CT192_{82\text{-}231}$-GFP-$Pep12_{L\text{-}TM}$; *ct192*$_{82\text{-}231}$ was amplified from pJB49 by PCR with oligos 2052 and 2053 and inserted by restriction-free cloning in pJB55.	This work
pJB83	$CT324_{119\text{-}303}$-GFP-$Pep12_{L\text{-}TM}$; *ct324*$_{119\text{-}303}$ was amplified from pJB51 by PCR with oligos 2058 and 2059 and inserted by restriction-free cloning in pJB55.	This work
pJB84	$CT135_{1\text{-}209}$-GFP-$Pep12_{L\text{-}TM}$; *ct135*$_{1\text{-}209}$ was amplified from pJB47 by PCR with oligos 2050 and 2051 and inserted by restriction-free cloning in pJB55.	This work
pJB85	$CT556_{1\text{-}99}$-GFP-$Pep12_{L\text{-}TM}$; *ct556*$_{1\text{-}99}$ was amplified from pJB52 by PCR with oligos 2059 and 2060 and inserted by restriction-free cloning in pJB55.	This work
pJB86	$CT233_{1\text{-}99}$-GFP-$Pep12_{L\text{-}TM}$; *ct233*$_{1\text{-}99}$ was amplified from *C. trachomatis* L2/434 chromosomal DNA by PCR with oligos 2065 and 2066 and inserted by restriction-free cloning in pJB55.	This work
pJB87	$CT179_{53\text{-}170}$-GFP-$Pep12_{L\text{-}TM}$; *ct179*$_{53\text{-}170}$ was amplified from pJB54 by PCR with oligos 2069 and 2070 and inserted by restriction-free cloning in pJB55.	This work
pJB88	$CT119_{57\text{-}246}$-GFP-$Pep12_{L\text{-}TM;}$ *ct119*$_{57\text{-}246}$ was amplified from pJB43 by PCR with oligos 2164 and 2165 and inserted by restriction-free cloning in pJB55.	This work
	**Plasmids for production of proteins in mammalian cells**	
pALT1	mEGFP; pALT1 derivatives were used for ectopic production of CT006-mEGFP fusion proteins in mammalian cells (Km^R).	Pais *et al.*, 2019

S4 Table. ***Continued.***

Plasmid	Description	Source/Reference
pALT2	mEGFP; pALT2 derivatives were used for ectopic production of mEGFP-CT006 fusion proteins in mammalian cells (Km^R)	Pais *et al.*, 2019
pJB104	mEGFP-$CT006_{FL}$; *ct006*$_{FL}$ was amplified from *C. trachomatis* L2/434 chromosomal DNA by PCR with oligos 2237 and 2238, digested with XhoI-BamHI and inserted in the same sites of pALT2.	This work
pJB105	mEGFP-$CT006_{1\text{-}88}$; *ct006*$_{1\text{-}88}$ was amplified from *C. trachomatis* L2/434 chromosomal DNA by PCR with oligos 2237 and 2241, digested with XhoI-BamHI and inserted in the same sites of pALT2.	This work
pJB106	$CT006_{FL}$-mEGFP; *ct006*$_{FL}$ was amplified from *C. trachomatis* L2/434 chromosomal DNA by PCR with oligos 2239 and 2263, digested with XhoI-BamHI and inserted in the same sites of pALT1.	This work
pJB107	$CT006_{1\text{-}88}$-mEGFP; *ct006*$_{1\text{-}88}$ was amplified from *C. trachomatis* L2/434 chromosomal DNA by PCR with oligos 2239 and 2242, digested with XhoI-BamHI and inserted in the same sites of pALT1.	This work
pJB110	$CT006_{139\text{-}189}$-mEGFP; *ct006*$_{139\text{-}189}$ was amplified from *C. trachomatis* L2/434 chromosomal DNA by PCR with oligos 2271 and 2263, digested with XhoI-BamHI and inserted in the same sites of pALT1.	This work
pJB113	mEGFP-$CT006_{139\text{-}189}$; *ct006*$_{139\text{-}189}$ was amplified from *C. trachomatis* L2/434 chromosomal DNA by PCR with oligos 2272 and 2238, digested with XhoI-BamHI and inserted in the same sites of pALT2.	This work
pJB140	mEGFP-$CT006_{1\text{-}88}$ with residues $H_{80}K_{81}$ substituted by $G_{80}G_{81}$; *ct006*$_{1\text{-}88}$ with nucleotide substitutions was generated by two distinct PCR reactions from pJB105 with oligos 2639 and 628, and oligos 2237 and 2640. The two resulting PCR products were used as templates to perform an overlapping PCR with oligos 2237 and 628. The final PCR product was digested with XhoI-BamHI and inserted in the same sites of pALT2.	This work
pJB141	mEGFP-$CT006_{1\text{-}88}$ with residues $R_{72}H_{80}K_{81}$ substituted by $G_{72}G_{80}G_{81}$; *ct006*$_{1\text{-}88}$ with nucleotide substitutions was generated by two distinct PCR reactions from pJB140 with oligos 2637 and 628, and oligos 2237 and 2638. The two resulting PCR products were used as templates to perform an overlapping PCR with oligos 2237 and 628. The final PCR product was digested with XhoI-BamHI and inserted in the same sites of pALT2.	This work
pJB142	mEGFP-$CT006_{1\text{-}88}$ with residues $K_{34}K_{37}H_{80}K_{81}$ substituted by $G_{34}G_{37}G_{80}G_{81}$; *ct006*$_{1\text{-}88}$ with nucleotide substitutions was generated by two distinct PCR reactions from pJB140 with oligos 2635 and 628, and oligos 2237 and 2636. The two resulting PCR products were used as templates to perform an overlapping PCR with oligos 2237 and 628. The final PCR product was digested with XhoI-BamHI and inserted in the same sites of pALT2.	This work

S4 Table. ***Continued.***

Plasmid	Description	Source/Reference
pJB143	mEGFP-CT006$_{1\text{-}88}$ with residues $K_{34}K_{37}R_{72}H_{80}K_{81}$ substituted by $G_{34}G_{37}G_{72}G_{80}G_{81}$; *ct006$_{1\text{-}88}$* with nucleotide substitutions was generated by two distinct PCR reactions from pJB141 with oligos 2635 and 628, and oligos 2237 and 2636. The two resulting PCR products were used as templates to perform an overlapping PCR with oligos 2237 and 628. The final PCR product was digested with XhoI-BamHI and inserted in the same sites of pALT2.	This work
pJB144	mEGFP-CT006$_{1\text{-}88}$ with residues $K_{34}K_{37}$ substituted by $G_{34}G_{37}$; *ct006$_{1\text{-}88}$* with nucleotide substitutions was generated by two distinct PCR reactions from pJB105 with oligos 2635 and 628, and oligos 2237 and 2636. The two resulting PCR products were used as templates to perform an overlapping PCR with oligos 2237 and 628. The final PCR product was digested with XhoI-BamHI and inserted in the same sites of pALT2.	This work
	**Plasmids for production of proteins in *C. trachomatis***	
p2TK2--SW2	*C. trachomatis* vector for expression of proteins (Amp^R).	Agaisse *et al.*, 2013
pSVP247 Derivatives	pSVP247 derivatives were used for production of proteins with a carboxy-terminal double HA (2HA) tag in *C. trachomatis*. Contain the terminator of the *incDEFG* operon (T*incD*) of *C. trachomatis* L2/434 (Amp^R).	da Cunha *et al.*, 2014
pAV4/pCT006-2HA	CT006-2HA; *ct006* and its predicted endogenous promoter were amplified from *C. trachomatis* L2/434 chromosomal DNA by PCR with oligos 2250 and 2251, digested with KpnI-NotI and inserted in the same sites of pSVP247.	This work
pJB134/pCT449-2HA	CT449-2HA; *ct449* and its predicted endogenous promoter were amplified from *C. trachomatis* L2/434 chromosomal DNA by PCR with oligos 2616 and 2617, digested with KpnI-NotI and inserted in the same sites of pSVP247.	This work
pJB151/pCT006$_{5G}$-2HA	CT006$_{5G}$-2HA: CT006-2HA with residues $K_{34}K_{37}R_{72}H_{80}K_{81}$ substituted by $G_{34}G_{37}G_{72}G_{80}G_{81}$; *ct006* with nucleotide substitutions was generated by several PCR reactions. Firstly, PCR product (PP) 1 was generated from pAV4 with oligos 2250 and 2663; PP2 was generated from pJB143 with oligos 2662 and 2665 and PP3 was generated from pAV4 with oligos 2664 and 2251. PP1 and PP2 and were then used as templates to perform an overlapping PCR with oligos 2250 and 2665, generating PP4. Finally, PP2 and PP4 were used as templates to perform an overlapping PCR with oligos 2250 and 2251 and this final PCR product was digested with NotI-KpnI and inserted in the same sites of pSVP247	This work

S4 Table. ***Continued.***

Plasmid	Description	Source/Reference
pSVP277	pSVP277 is a derivative of p2TK2--SW2 for production of proteins with a carboxy-terminal GSK peptide and contains the terminator of the *incDEFG* operon (T*incD*) of *C. trachomatis* L2/434.	This work
pSVP284	RplJ-GSK. The gene encoding RplJ-GSK is expressed under the control of the promoter of *ct694.*	This work
pSVP302/pTet-CteG-2HA	CteG-2HA. The gene encoding CteG-2HA is expressed under the control of the tetracycline-inducible promoter (P*tet*).	Pais *et al.*, 2019
pIP12	pIP12 is a derivative of pSVP277 without the first 3 nucleotides (ATG) of the DNA sequence encoding the GSK peptide and contains the terminator of the *incDEFG* operon (T*incD).* The DNA sequence from the gene encoding the GSK peptide (without the first ATG) to the end of the T*incD* was amplified by PCR from pSVP277 with oligos 2132 and 1483, digested with NotI-SalI and inserted in the same sites of p2TK2--SW2.	This work
pIP13/pTet-CteG-GSK	CteG-GSK; The DNA sequence encoding CteG under the control of the tetracycline-inducible promoter (P*tet*) was amplified by PCR from pSVP302 with oligos 1803 and 1552, digested with NotI-KpnI and inserted in the same sites of pIP12.	This work
pIP14/pRplJ-GSK	RplJ-GSK; The DNA sequence encoding RplJ under the control of the tetracycline-inducible promoter (P*tet*) was generated by two PCR reactions. A DNA fragment containing P*tet* was amplified by PCR from pSVP302 with oligos 1803 and 1808, and a DNA fragment encoding RplJ was amplified from pSVP284 with oligos 1809 and 1756. The two resulting PCR products were used as templates to perform an overlapping PCR with oligos 1803 and 1756. The final PCR product was digested with NotI-KpnI and inserted in the same sites of pIP12.	This work
pJB166/pCT006-GSK	CT006-GSK; *ct006* and its predicted endogenous promoter were amplified by PCR from pAV4 with oligos 2250 and 2251, digested with KpnI-NotI and inserted in the same sites of pIP12.	This work
pJB167	A DNA sequence containing the terminator of the *incDEFG* operon (T*incD)* was amplified by PCR from pIP12 with oligos 2778 and 1483, digested with NotI-SalI and inserted in the same sites of p2TK2--SW2.	This work

S4 Table. ***Continued.***

Plasmid	Description	Source/Reference
pJB168/pCT006-GSK(26)	CT006-GSK(26): GSK peptide fused to CT006 between amino acid residues 26 and 27. A DNA fragment encoding CT006-GSK(26) under the control of the *ct006* promoter was generated by sequential PCRs. A partial DNA fragment was amplified by PCR from pAV4 using oligos 2250 and 2781, and another DNA fragment was amplified with oligos 2780 and 2779. These PCR products were used as templates to perform an overlapping PCR with oligos 2250 and 2779. This final PCR product was digested with NotI-KpnI and inserted in the same sites of pJB167.	This work
pJB169/pCT006-GSK(39)	CT006-GSK(39): GSK peptide fused to CT006 between amino acid residues 39 and 40. A DNA fragment encoding CT006-GSK(39) under the control of the *ct006* promoter was generated by sequential PCRs. A DNA fragment was amplified by PCR from pAV4 using oligos 2250 and 2783, and another DNA fragment was amplified with oligos 2782 and 2779. These PCR products were used as templates to perform an overlapping PCR with oligos 2250 and 2779. These final PCR product was digested with NotI-KpnI and inserted in the same sites of pJB167.	This work
pJB170/pCT006$_{\Delta 47-67}$-2HA	CT006$_{\Delta 47-67}$-2HA: CT006-2HA lacking 21 amino acids from a putative hydrophobic domain (from amino acid residues A_{47} to V_{67}); *ct006*$_{\Delta 47-67}$ was generated by two PCR reactions from pAV4 with oligos 2250 and 2788, and oligos 2787 and 2251, followed by a PCR reaction where the two resulting PCR products were used as templates to perform an overlapping PCR with oligos 2250 and 2251. The final PCR product was digested with NotI-KpnI and inserted in the same sites of pSVP247.	This work

S4 Table References

Agaisse H, Derré I. A C. trachomatis cloning vector and the generation of C. trachomatis strains expressing fluorescent proteins under the control of a C. trachomatis promoter. PLoS One. 2013;8(2):e57090.

Da Cunha M, Milho C, Almeida F, Pais S V., Borges V, Maurício R, et al. Identification of type III secretion substrates of Chlamydia trachomatis using Yersinia enterocolitica as a heterologous system. BMC Microbiol. 2014;14:40.

De Felipe KS, Glover RT, Charpentier X, Anderson OR, Reyes M, Pericone CD, et al. Legionella eukaryotic-like type IV substrates interfere with organelle trafficking. PLoS Pathog. 2008;4(8):e1000117

Franco IS, Shohdy N, Shuman HA. The Legionella pneumophila effector VipA is an actin nucleator that alters host cell organelle trafficking. PLoS Pathog. 2012;8(2):e1002546.

Khaddaj R, Mari M, Cottier S, Reggiori F, Schneite R. The surface of lipid droplets constitutes a barrier for endoplasmic reticulum-resident integral membrane proteins. J Cell Sci. 2022;135(5):jcs256206.

Pais S V., Key CE, Borges V, Pereira IS, Gomes JP, Fisher DJ, et al. CteG is a Chlamydia trachomatis effector protein that associates with the Golgi complex of infected host cells. Sci Rep. 2019;9(1):6133.

Sisko JL, Spaeth K, Kumar Y, Valdivia RH. Multifunctional analysis of Chlamydia-specific genes in a yeast expression system. Mol Microbiol. 2006;60(1):51–66.
