# Supplementary material for: The Chlamydia trachomatis inclusion membrane protein CT006 associates with lipid droplets in eukaryotic cells
Source: PLoS One. 2022 Feb 22;17(2):e0264292. doi: 10.1371/journal.pone.0264292 (PMC8863265; doi:10.1371/journal.pone.0264292)
Supplement: S5 Table — (PDF) [file pone.0264292.s021.pdf]

S5 Table. Oligonucleotides used in this work.

Number	Description	Sequence (5' - 3')	Restriction Site
628	Reverse oligo to construct pJB140, pJB141, pJB142, pJB143 and pJB144.	TTATGTTTCAGGTTCAGGG	-
1483	Reverse oligo to construct pSVP277 and pIP12.	GATCGTCGACGTCTTAGGAGCTTT TTGCAATGC	SalI
1552	Reverse oligo to construct pIP13.	GATCGCGGCCGCGGATAGAGGAG CTTTGCACACC	NotI
1756	Reverse oligo to construct pIP14.	GATCGCGGCCGCGCTCTTGAGTT TTTTCTGCTTT CTGG	NotI
1803	Forward oligo to construct pIP13 and pIP14.	GATCGGTACCTTAAGACCCACTTT CACATTTAA	KpnI
1808	Reverse overlap oligo to construct pIP14.	GCAACTTTTTCTCTTCTTTCATTTC ACTTTTCTCTATCACTGATAGGGA GTGG	-
1809	Forward overlap oligo to construct pIP14.	CCACTCCCTATCAGTGATAGAGAA AAGTGAAATGAAAGAAGAGAAAAA GTTGC	-
1977	Forward oligo to construct pJB27.	AAAAGGATCCATGGGTATCAAACC TCATG	BamHI
1978	Reverse oligo to construct pJB27.	GGGGAAGCTTGCGAGCAACTTTT GCTGC	HindIII
1979	Forward oligo to construct pJB28.	AAAAGGATCCATGGCTTGTTGCGC ATGTG	BamHI
1980	Reverse oligo to construct pJB28.	GGGGAAGCTTTTCAGCACGGCTTT CTGTG	HindIII
1981	Forward oligo to construct pJB29.	AAAAGGATCCATGGCAGCAACGG TACCC	BamHI
1982	Reverse oligo to construct pJB29.	GGGGAAGCTTACTTGCACGAGCT CTTTTAAG	HindIII
1983	Forward oligo to construct pJB30 by restriction-free cloning.	GTCAAGGAGAAAAAACCCCGGAT CCATGTCTGGCTATGGTGGAGA	-
1984	Reverse oligo to construct pJB30 by restriction-free cloning.	CAGTGAAAAGTTCTTCTCCTTTACT CATAAGCTTATCATTGGGAAAAATT GAGTGTAGA	-
1985	Forward oligo to construct pJB31 by restriction-free cloning.	GTCAAGGAGAAAAAACCCCGGAT CCATGCGTCTTATGTATCGATCCT C	-
1986	Reverse oligo to construct pJB31 by restriction-free cloning.	CAGTGAAAAGTTCTTCTCCTTTACT CATAAGCTTAGAAGCTTGGTTAGC GTCTATA	-
1987	Forward oligo to construct pJB32 by restriction-free cloning.	GTCAAGGAGAAAAAACCCCGGAT CCATGAGAGCACGAAGTCGTC	-
1988	Reverse oligo to construct pJB32 by restriction-free cloning.	CAGTGAAAAGTTCTTCTCCTTTACT CATAAGCTTTTTTTTACGACGGGA TGCCT	-
1998	Forward oligo to construct pJB35.	GATCGGATCCATGACCCACCTTTT CCC	BamHI
1999	Reverse oligo to construct pJB35.	GATCAAGCTTAGCGGAAAAGCGTT GG	HindIII

S5 Table. ***Continued.***

Number	Description	Sequence (5' - 3')	Restriction Site
2000	Forward oligo to construct pLJM1076.	GATCGGATCCATGCAGCATGCCC ATAATG	BamHI
2001	Reverse oligo to construct pLJM1076.	GATCAAGCTTTTTTGTGTGCACTT GTGGC	HindIII
2002	Forward oligo to construct pJB33.	GATCGGATCCATGATCTGCCAGC GCAAC	BamHI
2003	Reverse oligo to construct pJB33.	GATCAAGCTTCTCTATACGCGCAT CTAAAGG	HindIII
2004	Forward oligo to construct pLJM1077.	GATCGGATCCATGTCTGTGATACG CAATTC	BamHI
2005	Reverse oligo to construct pLJM1077.	GATCAAGCTTATCCCACCCATGAA ATTTAGC	HindIII
2006	Forward oligo to construct pJB36.	GATCGGATCCATGTATGGGTTTTC TTTAAAACCG	BamHI
2007	Reverse oligo to construct pJB36.	GATCAAGCTTTCTCAGACTTTCTTC CAATAC	HindIII
2008	Forward oligo to construct pLJM1078.	GATCGGATCCATGTGTAGTCGGG GATTGC	BamHI
2009	Reverse oligo to construct pLJM1078.	GATCAAGCTTTGAGACACTTATAG TCACATCTGC	HindIII
2010	Forward oligo to construct pJB37.	GATCGGATCCATGGTCAAAGCCG CTCATC	BamHI
2011	Reverse oligo to construct pJB37.	GATCAAGCTTCTTCCAGTTTCTTTG TAAAAAGTCTCCG	HindIII
2012	Forward oligo to construct pJB40.	GATCGGATCCATGTTCGGATCTAT CCCTTG	BamHI
2013	Reverse oligo to construct pJB40.	GATCAAGCTTGCAATGAACACGAG CGC	HindIII
2014	Forward oligo to construct pJB34.	GATCGGATCCATGCGGATCTCTCA AAAAGATACTC	BamHI
2015	Reverse oligo to construct pJB34.	GATCAAGCTTGTGGCCGCGCTGG TTTTTC	HindIII
2016	Forward oligo to construct pLJM1079.	GATCGGATCCATGGAAGGATACTG TTCTCCG	BamHI
2017	Reverse oligo to construct pLJM1079.	GATCAAGCTTTTGGGTCTGATCCA CCAG	HindIII
2018	Forward oligo to construct pJB38.	GATCGGATCCATGAAATTACCAGA AGTGAG	BamHI
2019	Reverse oligo to construct pJB38.	GATCAAGCTTTTGCACCCAATTCA TTGAC	HindIII
2020	Forward oligo to construct pJB41.	GATCGGATCCATGCAAGTTGAGAA ATCTCAATG	BamHI
2021	Reverse oligo to construct pJB41.	GATCAAGCTTTATCGAACCACGTC TTCC	HindIII
2022	Forward oligo to construct pJB42.	GATCGGATCCATGTGGGATGAAG ACAGTTTG	BamHI
2023	Reverse oligo to construct pJB42.	GATCAAGCTTAATACCTGAGAATT GCCACC	HindIII

S5 Table. ***Continued.***

Number	Description	Sequence (5' - 3')	Restriction Site
2024	Forward oligo to construct pJB43.	GATCGGATCCATGCTACAGAAAAC CGCTAATC	BamHI
2025	Reverse oligo to construct pJB43.	GATCAAGCTTGGAGCTTTTTGTAG AGGG	HindIII
2026	Forward oligo to construct pJB39.	GATCGGATCCATGAGAGAGCGGT TGCATC	BamHI
2027	Reverse oligo to construct pJB39.	GATCAAGCTTGCTCGCCCCTTTTT TACTC	HindIII
2028	Forward oligo to construct pJB44.	GATCGGATCCATGGATGTTCTAGA GAATCATGG	BamHI
2029	Reverse oligo to construct pJB44.	GATCAAGCTTTTGAGTTACTAAAAT CACTTGGTCTG	HindIII
2030	Forward oligo to construct pJB45.	GATCGGATCCATGTCAGCAGTTGT TCAG	BamHI
2031	Reverse oligo to construct pJB45.	GATCAAGCTTGAAGGAGCGTGATC GAGAAC	HindIII
2046	Forward oligo to construct pJB55 by restriction-free cloning.	ACTATACAAAGTCGACCGATGCCC TATGTCGGAAGACGAATTTTTTGG	-
2047	Reverse oligo to construct pJB55 by restriction-free cloning.	GCTGACTGGGTTGAAGGCTCTCAT TACAATTTCATAATGAGAAAAATAA AAAG	-
2048	Forward oligo to construct pJB46 and pJB81 by restriction-free cloning.	GTCAAGGAGAAAAAACCCCGGAT CCATGCCCTCCACTGTTGCAC	-
2049	Reverse oligo to construct pJB46 and pJB81 by restriction-free cloning.	CAGTGAAAAGTTCTTCTCCTTTACT CATAAGCTTTCCGGAGCAACCCTG AAAG	-
2050	Forward oligo to construct pJB47 and pJB84 by restriction-free cloning.	GTCAAGGAGAAAAAACCCCGGAT CCATGGTAAGCTTCGATTTAAATG ATC	-
2051	Reverse oligo to construct pJB47 and pJB84 by restriction-free cloning.	CAGTGAAAAGTTCTTCTCCTTTACT CATAAGCTTTGTGATCATTTTGTTT CTTAATTTTC	-
2052	Forward oligo to construct pJB49 and pJB82 by restriction-free cloning.	GTCAAGGAGAAAAAACCCCGGAT CCATGTGTCACATCCGAAGCAG	-
2053	Reverse oligo to construct pJB49 and pJB82 by restriction-free cloning.	CAGTGAAAAGTTCTTCTCCTTTACT CATAAGCTTACAATCATTGGAAAC TAAATCATTAATC	-
2054	Forward oligo to construct pJB50 by restriction-free cloning.	GTCAAGGAGAAAAAACCCCGGAT CCATGGAGCACTACTCTCGTATTT G	-
2055	Reverse oligo to construct pJB48 and pJB50 by restriction-free cloning.	CAGTGAAAAGTTCTTCTCCTTTACT CATAAGCTTCACCCGAGAGCCGTA ATTG	-

S5 Table. ***Continued.***

Number	Description	Sequence (5' - 3')	Restriction Site
2056	Forward oligo to construct pJB48 by restriction-free cloning.	GTCAAGGAGAAAAAACCCCGGATCCATGTGTAGTTGTTGCTTGAGATC	-
2057	Forward oligo to construct pJB51 and pJB83 by restriction-free cloning.	GTCAAGGAGAAAAAACCCCGGATCCATGCTAGATAAAGAAAACAAATACC	-
2058	Reverse oligo to construct pJB51 and pJB83 by restriction-free cloning.	CAGTGAAAAGTTCTTCTCCTTTACTCATAAGCTTTTGTTCAATAACTCGTGTTGGAC	-
2059	Forward oligo to construct pJB52 and pJB85 by restriction-free cloning.	GTCAAGGAGAAAAAACCCCGGATCCATGCCATTCGCAAAAGAAGC	-
2060	Reverse oligo to construct pJB52 and pJB85 by restriction-free cloning.	CAGTGAAAAGTTCTTCTCCTTTACTCATAAGCTTATATTGTAGCCAGTCCCACTG	-
2065	Forward oligo to construct pJB86 by restriction-free cloning.	GTCAAGGAGAAAAAACCCCGGATCCATGACGTACTCTATGTCCGATATAG	-
2066	Reverse oligo to construct pJB86 by restriction-free cloning.	CAGTGAAAAGTTCTTCTCCTTTACTCATAAGCTTCTGCGGGCCGAGTCCTAAAG	-
2069	Forward oligo to construct pJB54 and pJB87 by restriction-free cloning.	GTCAAGGAGAAAAAACCCCGGATCCATGAAAGAACAAAAGCTTGCAGC	-
2070	Reverse oligo to construct pJB54 and PJB87 by restriction-free cloning.	CAGTGAAAAGTTCTTCTCCTTTACTCATAAGCTTACCTTTGGGAGAAGTATTTGCT	-
2132	Forward oligo to construct pIP12.	GATCGCGGCCGCAGTGGTCGCCCTCGCACTACTAGTTTCG	NotI
2162	Forward oligo to construct pJB71 by restriction-free cloning.	GTCAAGGAGAAAAAACCCCGGATCCATGGAAGGATACTGTTCTCCG	-
2163	Reverse oligo to construct pJB71 by restriction-free cloning.	CAGTGAAAAGTTCTTCTCCTTTACTCATAAGCTTTTGGGTCTGATCCACCAGAC	-
2164	Forward oligo to construct pJB88 by restriction-free cloning.	GTCAAGGAGAAAAAACCCCGGATCCATGCTACAGAAAACCGCTAATCTAC	-
2165	Reverse oligo to construct pJB88 by restriction-free cloning.	CAGTGAAAAGTTCTTCTCCTTTACTCATAAGCTTGGAGCTTTTTGTAGAGGGTGATG	-
2237	Forward oligo to construct pJB104 and pJB105.	GATCCTCGAGAACCCTCCACTGTTGCACC	XhoI
2238	Reverse oligo to construct pJB104 and pJB113.	GATCGGATCCTTAAGCGGAAAAGCGTTGG	BamHI
2239	Forward oligo to construct pJB106 and pJB107.	GATCCTCGAGATGCCCTCCACTGTTGC	XhoI
2241	Reverse oligo to construct pJB105.	GATCGGATCCTTATCCGGAGCAACCCTG	BamHI
2242	Reverse oligo to construct pJB107.	GATCGGATCCTCCGGAGCAACCCTGAAAG	BamHI

S5 Table. ***Continued.***

Number	Description	Sequence (5' - 3')	Restriction Site
2250	Forward oligo to construct pAV4.	GATCGGTACCACAATCAGCTTATC TCCCTAG	KpnI
2251	Reverse oligo to construct pAV4.	GATCGCGGCCGCGAGCGGAAAAG CGTTGGG	NotI
2263	Reverse oligo to construct pJB106 and pJB110.	GATCGGATCCAAAGCGGAAAAGC GTTGGG	BamHI
2264	Reverse oligo to construct pJB107.	GATCGGATCCAATCCGGAGCAAC CCTGAAAG	BamHI
2271	Forward oligo to construct pJB110.	GATCCTCGAGATGACCCACCTTTT CCCAGC	XhoI
2272	Forward oligo to construct pJB113.	GATCCTCGAGAAACCCACCTTTTC CCAGC	XhoI
2616	Forward oligo to construct pJB134.	GATCGGTACCATAGATAATGATTA TTATCAAG	KpnI
2617	Reverse oligo to construct pJB134.	GATCGCGGCCGCGCTGAATAGGC GCTTCAG	NotI
2635	Forward overlap oligo to construct pJB142, pJB143 and pJB144.	CCTCTAGCTCAAGGATACCCTGGA GCAGCATTATCCATAG	-
2636	Reverse overlap oligo to construct pJB142, pJB143 and pJB144.	CTATGGATAATGCTGCTCCAGGGT ATCCTTGAGCTAGAGG	-
2637	Forward overlap oligo to construct pJB141.	GTATTTCCTATCGGAGGTCTGATC TCCTGC	-
2638	Reverse overlap oligo to construct pJB141.	GCAGGAGATCAGACCTCCGATAG GAAATAC	-
2639	Forward overlap oligo to construct pJB140 .	GATCTCCTGCCTATTCGGTGGAAG CTTTCAGGGTTGC	-
2640	Reverse overlap oligo to construct pJB140.	GCAACCCTGAAAGCTTCCACCGAA TAGGCAGGAGATC	-
2662	Forward overlap oligo to construct pJB151.	CATAACACCTAGTTGGAAAAATGC CCTCCACTGTTGCACC	-
2663	Reverse overlap oligo to construct pJB151.	GGTGCAACAGTGGAGGGCATTTTT CCAACTAGGTGTTATG	-
2664	Forward overlap oligo to construct pJB151.	GCTTTCAGGGTTGCTCCGGATATG TTCTAGCAACCTTTC	-
2665	Reverse overlap oligo to construct pJB151.	GAAAGGTTGCTAGAACATATCCGG AGCAACCCTGAAAGC	-
2778	Forward oligo to construct pJB167.	GATCGCGGCCGCGGATGACATGT GATTCGCG	NotI
2779	Reverse oligo to construct pJB168 and pJB169.	GATCGCGGCCGCGTTAAGCGGAA AAGCGTTGGGAAGG	NotI
2780	Forward overlap oligo to construct pJB168.	GAAAGAGTTGCCGCTGCCAGTGG TCGCCCTCGCACTACTAGTTTCGC TGAAAGTTACATGAGCCCTCTAGC	-
2781	Reverse overlap oligo to construct pJB168.	GCTAGAGGGCTCATGTAACTTTCA GCGAAACTAGTAGTGCGAGGGCG ACCACTGGCAGCGGCAACTCTTTC	-
2782	Forward overlap oligo to construct pJB169.	CCCTAAAGCAGCAAGTGGTCGCC CTCGCACTACTAGTTTCGCTGAAA GTTTATCCATAGCCTCTTTAG	-

S5 Table. ***Continued.***

Number	Description	Sequence (5' - 3')	Restriction Site
2783	Reverse overlap oligo to construct pJB169.	CTAAAGAGGCTATGGATAAACTTT CAGCGAAACTAGTAGTGCGAGGG CGACCACTTGCTGCTTTAGGG	-
2787	Forward overlap oligo to construct pJB170.	CCATAGCCTCTTTAGCAGTATTTC CTATCAGAGG	-
2788	Reverse overlap oligo to construct pJB170.	CCTCTGATAGGAAATACTGCTAAA GAGGCTATGG	-
