# Supplementary material for: The Chlamydia trachomatis inclusion membrane protein CT006 associates with lipid droplets in eukaryotic cells
Source: PLoS One. 2022 Feb 22;17(2):e0264292. doi: 10.1371/journal.pone.0264292 (PMC8863265; doi:10.1371/journal.pone.0264292)
Supplement: Attachment — Submitted filename: Response to Reviewers.pdf [file pone.0264292.s022.pdf]

# <u>Point-by-point Response to the Academic Editor and Reviewers</u>

**PONE-D-21-38824**
**The *Chlamydia trachomatis* inclusion membrane protein L (IncL) associates with host cell lipid droplets**

## <u>Academic Editor</u>

**1 -** *"While both reviewers thought the experiments were well done, they also expressed concerns that the data does not support the overall conclusion that CT006 associates with lipid droplets during Chlamydia infection."*

**Response:** We agree that our data does not allow us to conclude that CT006 associates with lipid droplets <u>during *Chlamydia* infection</u>. To clarify this, we modified the title to "The *Chlamydia trachomatis* inclusion membrane CT006 associates with lipid droplets in eukaryotic cells" as well as in the remainder of the text whenever the writing could be ambiguous in this regard.

## Reviewer #1

**1 -** *"Missing citations:*
*Mital J (2013) PLOS One- Ectopically expressed several Incs*
*Weber MM (2015) Infect Immun- demonstrated that CT006 is a bona fide Inc"*

**Response:** These papers are now cited.

**2 -** *"Based on the data presented herein, I believe it is premature to rename CT006 to IncL. The observations that it might target lipid droplets largely relies on ectopic data and minimal infection experiments. Without a mutant to confirm it is important for lipid droplet recruitment or a binding partner, it is best to keep its designation as CT006."*

**Response:** we modified the manuscript throughout (text, figures, and supplementary data) replacing IncL by CT006.

**Reviewer #2**

**1 –** "*the authors have expressed CT229 FL or fragments in yeast and mammalian cells: CT229 FL is cytosolic, CT22991-215 is endosomal and CT2291-88 is targeted to LD. Thus positively charged sequences close to the amino-terminal hydrophobic domain CT229 (between aa 1 and 88) are likely responsible for the tropism of this peptide to LD – same situation with positively charged sequences of caveolin that mediate the sorting of caveolin to LD while central hydrophobic domain anchors of caveolin direct the protein to the ER. However, we cannot extrapolate from these data that CT229 physiologically interact with host LD in infected cells, more especially if CT229 FL does not localize to LD in yeast and mammalian cells.*"

**Response:** We agree that our data does not allow to extrapolate that CT006 (the protein in which we focused our studies) interacts with host LDs in infected cells. Please see Response to the Academic Editor.

**2 –** "*The localization of CT229 assessed by IFA in Chlamydia strains expressing IncL-2HA (with anti-HA) or GSK fused to IncL (with anti-GSK) is at the inclusion membrane from 16 to 24hpi (Fig. 6). In the attempt to examine whether CT229 is at least secreted into the host cell for LD interaction, the authors looked at CT229 localization at early time points (2 to 8hpi in Fig. S10), and observed a signal beyond the cytosolic signal of Hsp60. They conclude that CT229 is on extensions of the inclusion membrane. However, without showing a colocalization of CT229 with IncA or IncG that are expressed on extensions of the inclusion membrane, we cannot ascertain that CT229 are on extensions of the inclusion membrane. Irrespective to these data, CT229 is not observed close/around host LD: in Fig. 6. the CT229 red signal from IncL-2HA does not intersect with the green LD signal. The only conclusion of Fig. 6 is that few host LD are in proximity to the inclusion, at the limit of the fluorescence microscopy resolution (~200nm).*"

**Response:** We previously wrote "At 8 h post-infection IncL-2HA appeared in small tubules, which are likely an extension of the vacuolar membrane". To address the issue raised by the reviewer that "we cannot ascertain that CT229 are on extensions of the inclusion membrane", and as we do not have anti-IncA or anti-IncG antibodies, we now modified the text to "At 8 h post-infection CT006-2HA appeared in small tubules near the inclusion, suggesting that these tubules

could be an extension of the vacuolar membrane”. Being on the inclusion membrane, CT006, as any other Inc, can potentially interact with LDs on the host cell cytoplasm. We did not analyse a co-localization between CT006 and LDs by microscopy (our Fig. 6 shows CT006, Cap1 and chlamydial MOMP) because as CT006 is apparently homogenously distributed around the inclusion a normal overlap with some LDs would have little significance.

**3 –** *“The Discussion contains too many repetitions of the Results section (to be combined). What must be better discussed is the connection between CT229 with IncA (according to ref. 5, only IncA associate with purified host LD), IncX (in the proposed model in ref. 5) as well with IncG, CT618 and Cap1 (from ref. 10) in case of physiological role of CT229 on host LD.”*

**Response:** The discussion was abridged (~500 words) to avoid repetitions of the Results section, but we do prefer to keep the Discussion and Results as separate sections. We previously mentioned IncX (while not explicitly), and the previously shown association of IncA (in one study) and of IncG, CT618 and Cap1 (in another study) with LDs. From the existing data (ours and from these publications) we think the connection between CT006 and these proteins is not obvious and this is now clearly written.
